# Human IFIT proteins inhibit lytic replication of KSHV: A new feed-forward loop in the innate immune system

**DOI:** 10.1371/journal.ppat.1007609

**Published:** 2019-02-19

**Authors:** Dajiang Li, Sankar Swaminathan

**Affiliations:** 1 Division of Infectious Diseases, Department of Internal Medicine, University of Utah School of Medicine, Salt Lake City, Utah, United States of America; 2 George E. Wahlen Department of Veterans Affairs Medical Center, Salt Lake City, Utah, United States of America; UPMC Hillman Cancer Center, UNITED STATES

## Abstract

Kaposi’s sarcoma-associated herpesvirus (KSHV) is causally associated with Kaposi’s sarcoma, primary effusion lymphoma (PEL) and multicentric Castleman’s disease. The IFIT family of proteins inhibits replication of some viruses, but their effects on KSHV lytic replication was unknown. Here we show that KSHV lytic replication induces IFIT expression in epithelial cells. Depletion of IFIT1, IFIT2 and IFIT3 (IFITs) increased infectious KSHV virion production 25-32-fold compared to that in control cells. KSHV lytic gene expression was upregulated broadly with preferential activation of several genes involved in lytic viral replication. Intracellular KSHV genome numbers were also increased by IFIT knockdown, consistent with inhibition of KSHV DNA replication by IFITs. RNA seq demonstrated that IFIT depletion also led to downregulation of IFN β and several interferon-stimulated genes (ISGs), especially OAS proteins. OAS down-regulation led to decreased RNase L activity and slightly increased total RNA yield. IFIT immunoprecipitation also showed that IFIT1 bound to viral mRNAs and cellular capped mRNAs but not to uncapped RNA or trimethylated RNAs, suggesting that IFIT1 may also inhibit viral mRNA expression through direct binding. In summary, IFIT inhibits KSHV lytic replication through positively regulating the IFN β and OAS RNase L pathway to degrade RNA in addition to possibly directly targeting viral mRNAs.

## Author summary

The innate immune response to infections is triggered by recognition of pathogens as foreign or non-self. Recognition of invading pathogens is carried out by various sensors or pattern recognition receptors (PRRs) that detect conserved features of pathogens including lipids, nucleic acids and proteins. PRR activation triggers pathways that ultimately lead to pathogen destruction, including the interferon response. Interferons, in turn induce many interferon-stimulated genes, which inhibit or destroy a wide variety of pathogens, including viruses. IFITs are a family of interferon induced proteins that are thought to recognize RNAs and have antiviral effects primarily on RNA viruses. Kaposi’s sarcoma-associated herpesvirus (KSHV), a DNA virus, is associated with Kaposi’s sarcoma and lymphoid malignancies. In this study we show that IFITs restrict replication of KSHV and does so not only by inhibiting KSHV mRNA abundance but also by enhancing other effectors of the interferon response. This study reveals that the innate immune response can control not only invading viruses but ones that reactivate from latency, that IFITs can inhibit herpesvirus replication and that IFITs may amplify the innate immune response by a feed-forward mechanism.

## Introduction

Kaposi’s sarcoma-associated herpesvirus (KSHV, HHV8) is causally associated with Kaposi’s sarcoma (KS), primary effusion lymphoma (PEL) and multicentric Castleman’s disease (for a review, see reference [1]). KSHV maintains a persistent latent infection in B lymphocytes, from which it occasionally reactivates, enters a lytic cycle of replication, and produces infectious virions. Transmission occurs by both sexual and nonsexual contact as well as blood and organ transfusion. Cell-mediated immunity is important for limiting KSHV reactivation and pathogenesis. KSHV infection activates several pattern recognition receptors (PRRs), including cGAS, IFI16, RIG-I, NLRP1, and several Toll-like receptors (TLRs) which play an important role in promoting the innate immune response [2–7]. KSHV pathogen associated molecular patterns (PAMPs) recognized by the innate immune system remain to be fully characterized but are primarily thought to reside on viral glycoproteins and nucleic acids [8].

Much of the work done on the innate immune response to KSHV has used systems in which the cellular response to incoming virus has been examined. These studies have shown that cytoplasmic and endosomal viral nucleic acids may be detected by one or more PRRs [9] and that viral glycoproteins may activate PRRs upon viral entry [9–12]. Several DNA sensors may be important in recognition of viral DNA, including cyclic GMP synthetase (cGAS) and IFI16. IFI16 has recently been shown to act in the nucleus to activate a nuclear inflammasome in response to KSHV and EBV infection [2, 13, 14]. cGAS mediated recruitment of STING and IRF3 activation requires association with a ribonucleoprotein complex which is remodeled by foreign DNA [15]. Several components of these cytoplasmic innate immune pathways are involved in the innate immune response to KSHV. TLR9 appears to act as a sensor for incoming KSHV DNA and partly contributes to the activation of IFN-α [10]. Both NLRP1, a protein component of the inflammasome, and IFI16 may restrict KSHV reactivation, since depletion of NLRP1 or IFI16 results in increased lytic replication [3, 16]. Similarly, RIG-I, a cytosolic RNA sensor, may be important in limiting KSHV reactivation from latency as KSHV lytic replication was enhanced in RIG-I^-/-^ cells. The importance of these pathways is emphasized by the fact that KSHV counteracts the host response via vIRF-1, LANA and ORF52 [4, 6, 17].

The PAMPs displayed by herpesviruses are predicted to differ considerably depending on the stage and type of their replicative cycles. During latency, herpesviruses exist as chromatinized nuclear episomes. During stringent latency, few, if any, lytic proteins or RNAs are synthesized, and virion DNA is not produced [1]. In contrast, once reactivation occurs, and the virus enters the lytic replicative cycle, abundant amounts of viral mRNAs and non-coding RNAs are produced and newly replicated genomes are produced, encapsidated, and egress from the nucleus prior to tegumentation, final envelopment and transit through the plasma membrane. Thus, while virion DNA is not expected to be exposed to endosomes and cytoplasm in the same context as during primary infection of the cell, there is nevertheless ample of opportunity for virus components such as mRNAs, non-coding RNAs, and viral proteins to be detected by cytoplasmic PRRs and trigger innate immune responses. We therefore wished to extend our study of host cellular factors to cytoplasmic PRRs that could contribute to establishment of an antiviral state and restrict lytic KSHV replication and reactivation from latency. These include human IFIT proteins that have been recently demonstrated to play important roles in the inhibition of several viruses besides herpesviruses [18, 19].

The IFN induced tetratricopeptide repeat containing proteins (IFITs) are among the most highly interferon-induced proteins [20]. They constitute a family of related genes that have been identified in a wide variety of mammals from mouse to man [21]. The human genes, encoded on chromosome 10, are IFIT1 (ISG56), IFIT2 (ISG54), IFIT3, IFIT5 and IFIT1B [21]. Several of the IFITs have been implicated in an important antiviral response pathway dependent on recognition of foreign RNAs. In mice, Ifit1, a homolog of human IFIT1B, specifically recognizes uniquely modified viral RNAs that lack 2'O-methylation of their 5' mRNA caps (cap0-mRNAs) [22, 23]. Viruses that replicate in the cytoplasm that have either “snatched” a cap or encode their own 2”O-methyltransferase may thereby evade recognition as non-self [24]. In humans however, IFIT1 protein differs significantly from IFIT1B, and may play a broader role in antiviral function, with different RNA binding specificities [21]. IFIT1 forms a tripartite complex with IFIT2 and IFIT3 and binds to 5’ mRNA caps [25]. In addition to inhibiting replication of viruses that are predicted to have 2’O-methylated caps, IFIT1 inhibits papillomavirus replication by binding its E1A protein [26]. IFIT1 inhibits translation of viral mRNAs by preferentially binding their 5’ cap and preventing association with eukaryotic initiation factors [23]. In addition, IFIT1 has been shown to affect protein translation by interacting with eukaryotic initiation factor eIF3 and may thereby inhibit additional virus families by different mechanisms [18]. It was therefore of interest to determine whether IFIT proteins could inhibit KSHV, whose replication strategy differs considerably from other virus families in which IFITs have been shown to exert antiviral activity.

In this study, we examined the effect of IFIT proteins on KSHV replication by depleting IFITs under conditions whereby highly efficient KSHV lytic replication and infectious virion production was enabled in epithelial cells. KSHV lytic gene expression, DNA replication and virion production were enhanced by depletion of IFITs. Further, IFIT expression, which was undetectable during latent infection, was induced during the course of KSHV reactivation and lytic replication. Using deep sequencing of mRNA, we analyzed the effects of IFITs on KSHV and cellular transcript accumulation during lytic KSHV replication. In addition to IFIT effects on the viral transcriptome, we discovered an unexpected positive effect on the expression of other members of the interferon-induced response that is predicted to amplify the antiviral effect of IFIT proteins.

## Results

### KSHV lytic replication induces IFIT expression

**Fig 1:**
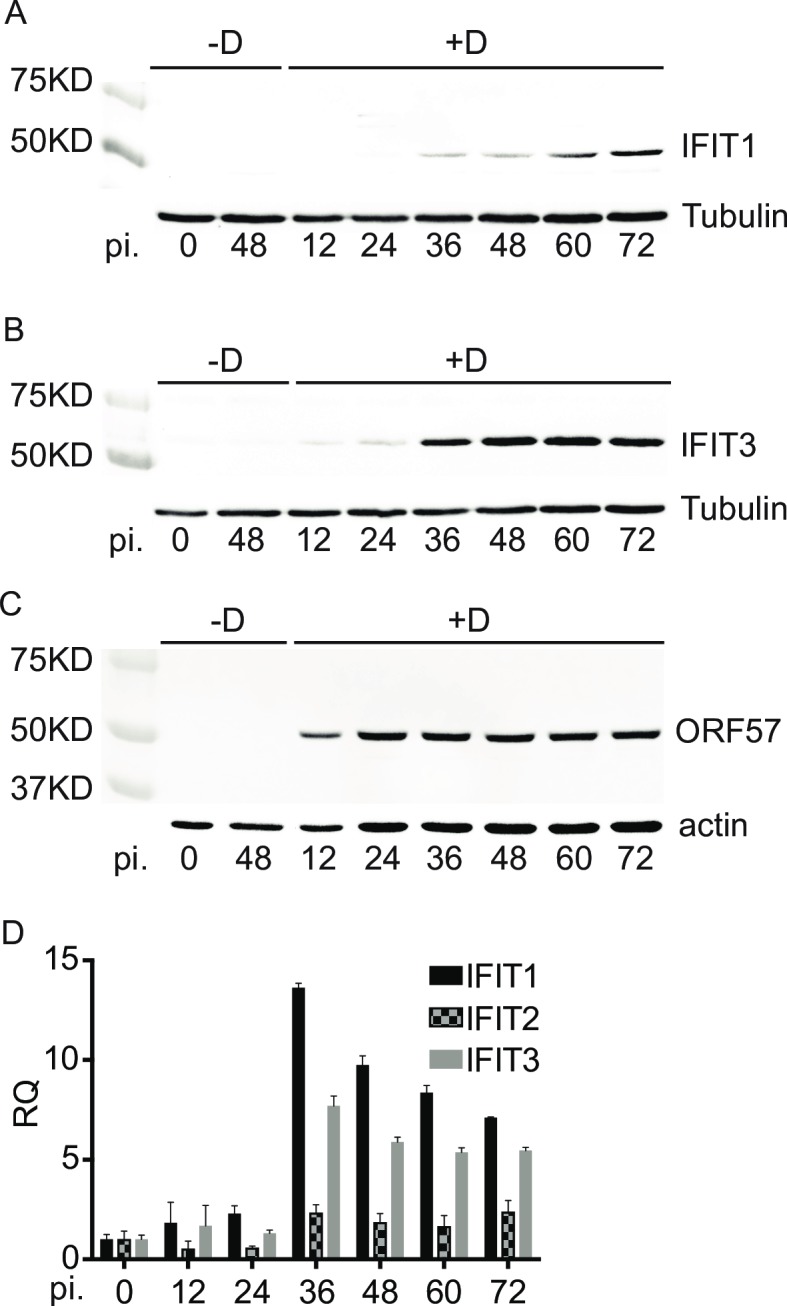
IFIT1 and IFIT3 expression is induced by KSHV lytic replication. KSHV-infected iSLK cells were untreated (-D) or treated with doxycycline (+D) to induce replication. Cells were harvested at different times post induction (pi) as shown. Immunoblotting of lysates from the cells was performed with anti-IFIT1, anti-IFIT3 and anti-ORF57 antibodies to measure IFIT1 (A), IFIT3 (B) and ORF57 (C) protein expression. Tubulin and actin blots are shown as a loading control. qPCR was performed to measure IFIT RNA expression in the samples from different time post induction as shown (D).

IFIT genes are strongly induced by several viral infections, including the betaherpesvirus hCMV [18, 19, 27]. Although several innate immune pathways are induced by KSHV infection or reactivation, it was not known whether IFITs were induced by KSHV lytic replication during reactivation from latency. We therefore examined the status of IFIT protein expression in iSLK/Bac16 cells stably transduced with a doxycycline-inducible viral transactivator, KSHV ORF50/Rta [59]. These Rta-inducible SLK cells (iSLK) are stably and latently infected with the Bac16 KSHV strain that expresses hygromycin resistance and GFP, and robust and synchronous reactivation of KSHV from latency is achieved by doxycycline treatment. [30]. Infected cells were 100% GFP positive when maintained under hygromycin selection (S1 Fig). iSLK/Bac16 cells were treated with doxycycline and cells were harvested at serial time points from 0–72 hrs at 12 hr intervals. We measured IFIT1 and IFIT3 protein levels in iSLK cells by Western blotting. Both IFIT1 and IFIT3 were not detectable in uninduced cells but were expressed after KSHV reactivation (Fig 1 and S2 Fig). IFIT1 protein was first detectable at 36hr post induction (p.i.) and continued to increase to 72 hr. IFIT3 was first detectable at 12 hr and reached peak expression by 48 hr. (Fig 1A and 1B). In order to confirm that exogenous Rta expression itself did not affect IFIT expression, we also assessed IFIT1 and IFIT3 expression in doxycycline treated or untreated uninfected iSLK cells. Neither IFIT1 and IFIT3 was detectable at 48 hr and 72 hr (S3 Fig). Induction of KSHV Bac16 lytic replication was confirmed by immunoblotting for ORF57 which was expressed by 12hr after induction (Fig 1C). qPCR also showed IFIT1, IFIT2 and IFIT3 expression peaking by 36hr which was consistent with the results of Western blotting (Fig 1D). These results clearly demonstrate that KSHV lytic replication induces IFIT expression.

### IFIT1 and IFIT3 induced by KSHV replication localize in the cytoplasm of infected cells

**Fig 2:**
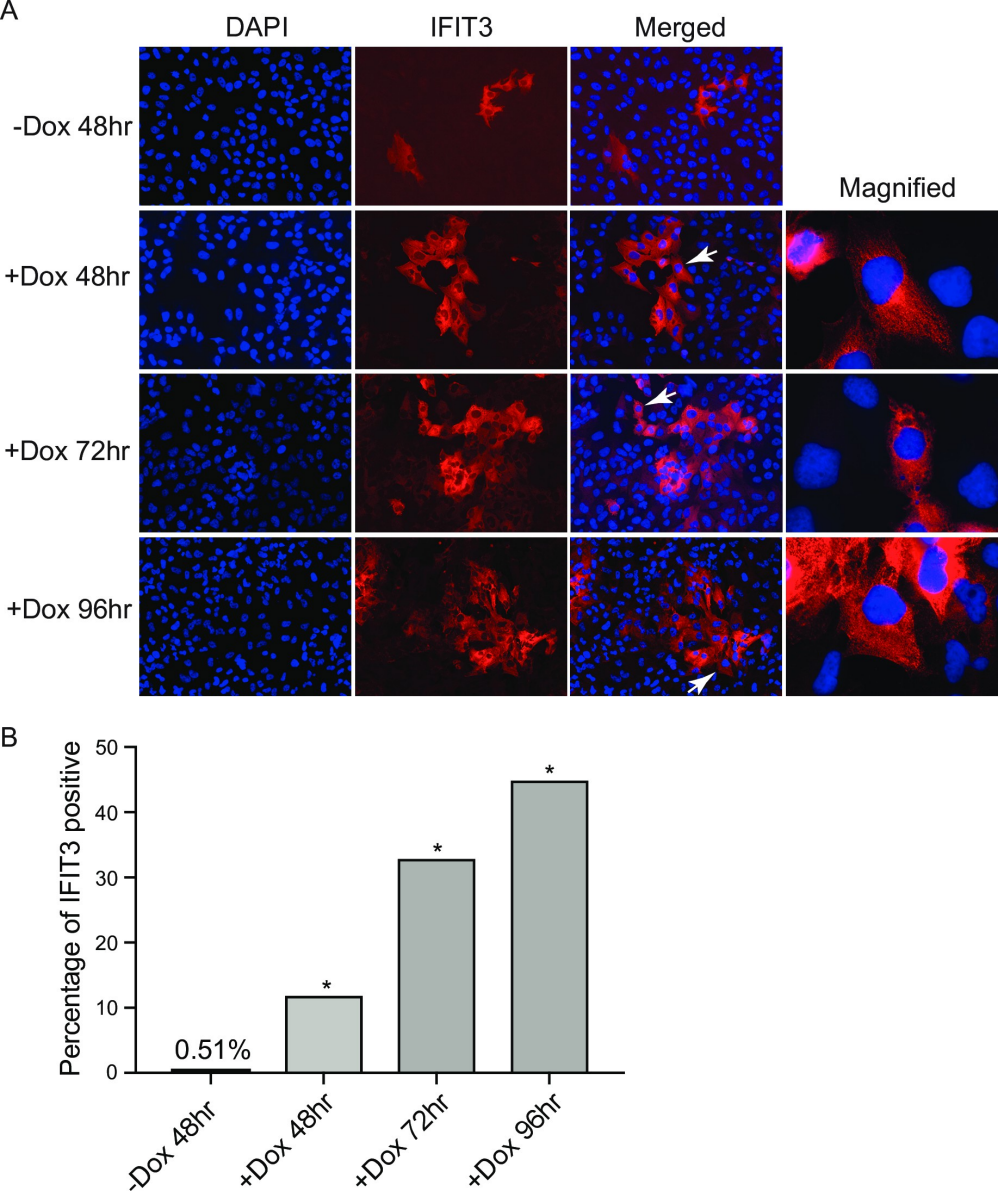
IFIT3 is induced by KSHV lytic replication and localizes to the cytoplasm of infected cells. A. Immunofluorescence microscopy of IFIT3 in KSHV infected cells induced to permit lytic replication. KSHV-infected iSLK cells were untreated (-D) or treated with doxycycline (+D) to induce replication. Cells were fixed at 48hr, 72hr and 96hr post induction (pi) as shown. Cells were then stained for IFIT3 (Red). Arrows indicate magnified cells which are shown at right in each panel. B. Percentages of IFIT3 positive staining cells were counted at different time point post induction (pi) as shown. *P < 0.0001.

In order to confirm induction of IFITs as a result of KSHV replication and to determine their cellular location, we examined lytically induced KSHV infected cells by immunofluorescence microscopy. iSLK cells were grown on glass coverslips and treated with 1 μg/ml doxycycline to induce virus lytic replication. Cells were fixed at 48hr, 72hr and 96hr post induction. Immunofluorescence staining for IFIT3 was performed and revealed cytoplasmic expression (Fig 2A). A small percentage of cells were IFIT3 positive before induction, possibly in cells which undergo spontaneous lytic gene expression. However, the percentage of cells expressing IFIT3 increased progressively after lytic induction, and was approximately 45-fold higher by 96 hrs (Fig 2B). These results confirmed the immunoblotting data and demonstrate that IFIT3 is expressed in the cytoplasm. IFIT1 exhibited similar cytoplasmic localization to IFIT3 (S4 Fig).

### IFIT depletion enhances lytic KSHV replication and virus production

**Fig 3:**
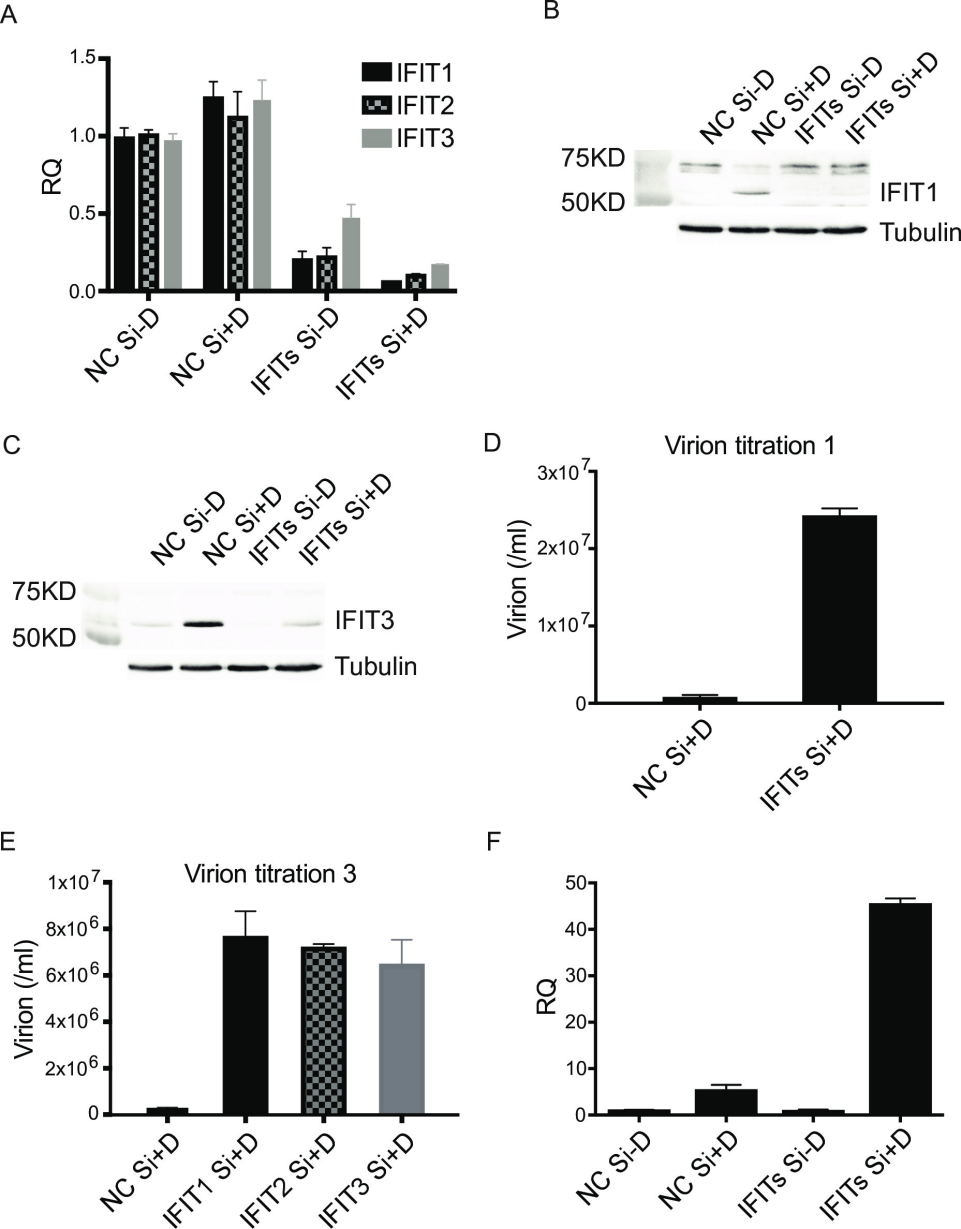
KSHV virus production in cells depleted of IFIT1, IFIT2 and IFIT3. A. Effect of IFIT KD on IFIT1, IFIT2 and IFIT3 mRNA levels. IFIT and negative control KDs were performed in iSLK cells and RNA was isolated from cell pellets 48 hr after induction of replication. IFIT1, IFIT2 and IFIT3 mRNA expression was measured by qPCR. Cells were either untreated (-D) or treated with doxycycline (+D) to induce replication. RQ (relative quantity). Each experiment was performed with biological triplicates and technical triplicates. Error bars show SEM of qPCR from triplicate samples. B, C. Effect of IFIT KD on IFIT protein levels. Immunoblotting of lysates from cells used in panel A above was performed with anti-IFIT1 and anti-IFIT3 antibodies to verify completeness of IFIT1 and IFIT3 depletion. Lysates were prepared from cells harvested at the time of replication induction with doxycycline. Immunoblots for tubulin are shown as loading controls. D. Effect of IFIT depletion on infectious virion production. KSHV-infected iSLK cells were transfected with either control siRNA (NC Si) or a mixture of IFIT1, IFIT2 and IFIT3-specific siRNA (IFITs Si) and KSHV replication was induced by treatment with doxycycline. Supernatants from induced cells were used to infect 293T cells, leading to GFP expression in infected cells. Infectious virus titer in the supernatants was measured by flow cytometry to quantitate GFP-positive 293T cells. Each transfection/induction was performed in triplicate and three replicate infections were performed with each supernatant. Error bars show SEM of titration from triplicate samples. E. Effect of individual IFIT depletion on infectious virion production. KSHV-infected iSLK cells were transfected with either control siRNA (NC Si) or IFIT1, IFIT2 or IFIT3-specific siRNA individually. KSHV replication was induced by treatment with doxycycline and infectious virus titration was performed as in D. Each transfection/induction was performed in triplicate and three replicate infections were performed with each supernatant. Error bars show SEM of titration from triplicate samples. F. Effect of IFIT depletion on KSHV DNA replication. Combined IFIT1, IFIT2 and IFIT3 KD and negative control KDs were performed in iSLK cells as in A. Cellular and viral DNA was isolated from cell pellets. Relative KSHV genome copy number was measured by qPCR. Each experiment was performed with biological triplicates and technical triplicates. Error bars show SEM of qPCR from triplicate samples.

In order to investigate IFIT1, IFIT2 and IFIT3’s effect on KSHV lytic replication and reactivation from latency, we measured virion production in KSHV infected cells (iSLK/Bac16) after depletion of IFITs. IFIT depletion was carried out by lipid-mediated transfection of iSLK cells with siRNA specific for IFITs (Fig 3A–3C). Cells were transfected with siRNAs 6 hr prior to inducing lytic replication. We first measured IFIT RNA abundance at 48 hr post induction by qPCR (Fig 3A). Approximately 90% depletion of IFIT mRNAs was apparent in cells induced to permit KSHV replication. We next examined expression of IFIT1 and IFIT3 by immunoblotting from samples harvested at 48 hr post induction. Approximately 90% depletion of IFIT1 protein was achieved by 48 hr post-transfection as assessed by densitometry of the Western blot (Fig 3B). There was approximately 73% depletion of IFIT3 (Fig 3C). In order to assess the effect of IFIT depletion on KSHV reactivation and virion production, cells were transfected with either IFIT siRNAs or control siRNA, and 6 hours later KSHV reactivation was induced by addition of doxycycline. Virion-containing supernatant was harvested at 120 hours after induction of lytic replication. Infectious virus production was measured by infection of 293T cells with serial dilutions of virus supernatant followed by flow cytometry of infected cells. Virus titer in the supernatant can thus be accurately quantitated as GFP-transducing units [29]. As shown in Fig 3D and S5 Fig (IFIT KD and virion titration repeated in a separate experiment), IFIT depletion led to a marked increase in virion production (25–32 fold), compared to control siRNA-transfected cells induced in parallel. There was no microscopically detectable release of infectious virus in the absence of doxycycline from either IFIT depleted cells or in control cells, indicating that Rta is still absolutely required for lytic replication. These data indicate that the IFITs act as a restriction factor for KSHV virus production.

IFIT1, IFIT2 and IFIT3 may form a tripartite complex and cooperate in RNA binding [23, 25, 30]. Therefore, we performed virion titration as was done in the previous experiments to examine the effect on virion production of individual depletion of each IFIT. Individual depletion of the three IFITs had a similar effect on virion release, with each IFIT depletion leading to a marked increase in virion production (about 22–25 fold) (Fig 3E). The magnitude of this effect is similar to that observed upon depletion of all three IFITs together. These data suggest that each of the three IFITs is important for restriction of KSHV virus production.

We next wished to ask at which stage of KSHV lytic replication IFITs might be exerting an inhibitory effect on KSHV virion production. To determine whether the IFIT effect was due to inhibition of KSHV DNA replication, we measured KSHV genome abundance by qPCR on DNA samples from cells that were induced to replicate after depletion or mock depletion of IFITs. The results demonstrated that intracellular KSHV genome copy numbers increased at least 9-fold upon IFIT KD (Fig 3F). However, this increase was not as large as the increases observed in infectious virus titer (Fig 3D, S5 Fig), suggesting that IFITs may affect other steps in the lytic KSHV cycle in addition to DNA replication.

### IFIT depletion broadly upregulates KSHV gene expression

**Fig 4:**
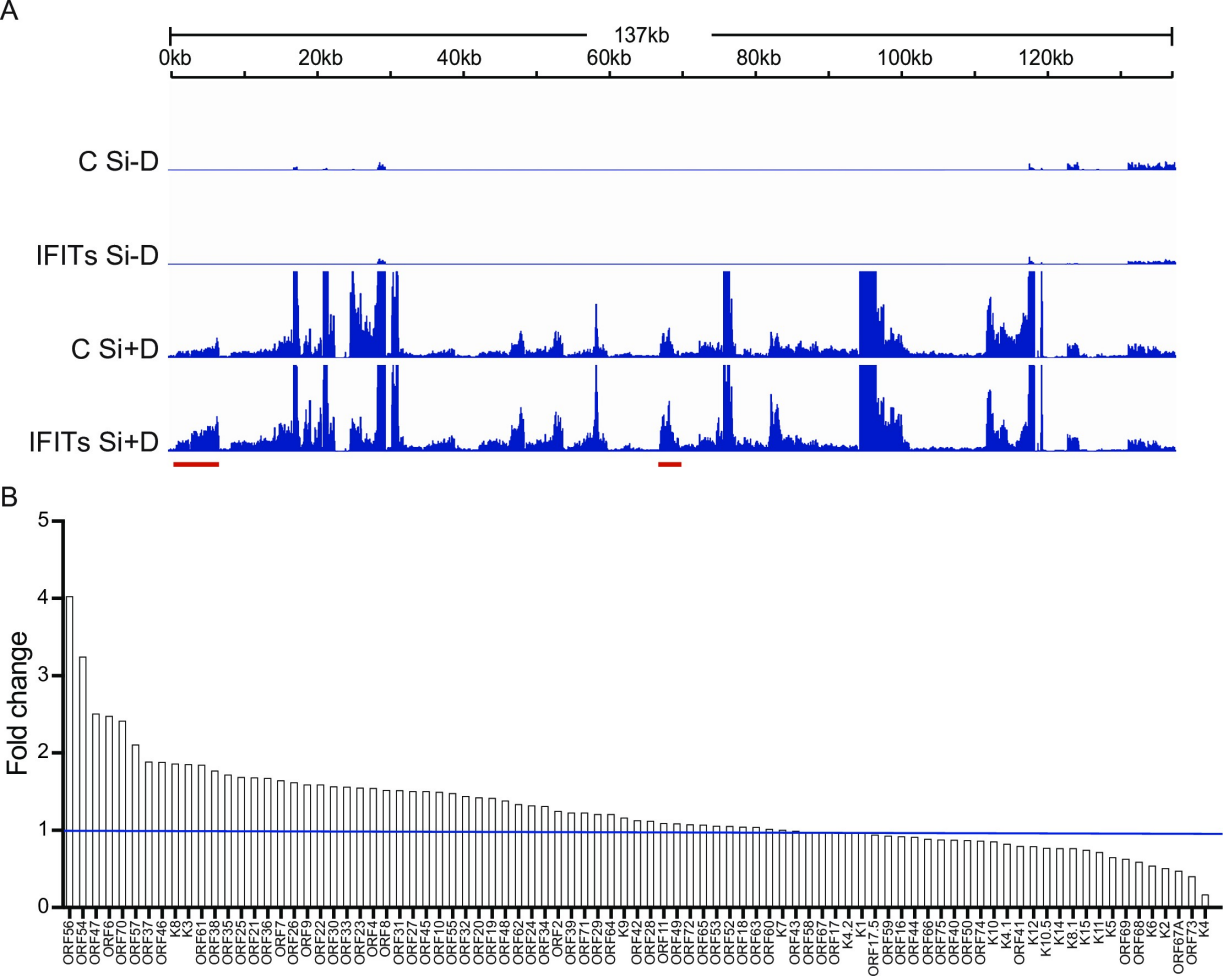
Effect of IFITs depletion on the KSHV lytic gene transcriptional profile defined by RNA-Seq. A. Transcriptome of KSHV in iSLK cells at 48 h after induction of lytic replication. iSLK cells were depleted of IFIT1, IFIT2 and IFIT3 (IFITs) with siRNAs or transfected with negative control siRNA. Cells were then either uninduced or induced to permit lytic KSHV replication by treatment with doxycycline. RNA was harvested at 48 hr from each sample and RNA sequencing was performed. The ratio of read number for each position on the KSHV genome in cells depleted of IFITs versus the values for corresponding control cells (NC) are shown on the y-axis and the KSHV genome position is shown on the x axis. Representative genes where IFITs knockdown led to increased transcription compared to control are marked below with red bars. B. Relative effect of IFITs depletion on KSHV mRNAs. The effect of IFITs depletion on each annotated KSHV transcript is depicted in a waterfall plot as the fold enrichment of its RNA abundance in the absence versus presence of IFITs at 48 hr after induction. Transcripts whose levels increase with IFITs knockdown are thus shown above 1 (1 marked as blue line) and transcripts that decrease in abundance with IFITs knockdown are shown below 1.

IFIT depletion enhanced KSHV virion production (25–32 fold) while KSHV DNA copy number increased only 9-fold, suggesting that IFITs may restrict expression of late genes that are needed for virion formation, egress or infectivity. In order to assess the global effect of IFITs on KSHV lytic gene expression, we performed high-throughput deep sequencing of mRNA from KSHV-infected cells in which IFITs were depleted prior to induction of lytic replication. KSHV-infected iSLK cells were transfected with either control siRNA or IFIT siRNAs as was done in the previous experiments to examine the effect on virion production. Six hours after siRNA transfection, cells were treated with doxycycline to induce KSHV lytic replication, and cells were harvested at 48 hours post induction, RNA was isolated, oligo-dT selected, and processed for deep sequencing. The effects of IFIT KD on lytic cycle transcription were compared to the transcriptional profile of induced cells transfected with control siRNA. A comparison of the transcriptional profiles is presented in Fig 4A. Consistent with its effect on virus production, IFIT KD was associated with broad enhancement of KSHV lytic gene expression. Approximately two thirds of genes demonstrated increased expression: 6 genes increased > 2-fold and 35 genes increased 1.2-2-fold. 30 genes did not exhibit increases (less than 20% change) while 15 genes decreased 20% -85% (Fig 4B). Of the six genes whose expression was increased more than two-fold, 5 are involved in lytic KSHV DNA replication: ORF56, the helicase primase; ORF54, the deoxyuridine triphosphatase; ORF6, the single-stranded DNA-binding protein; ORF70, thymidylate synthase; and ORF57, a post-transcriptional regulator that preferentially enhances mRNA accumulation of several genes involved in DNA replication [31]. The sixth gene ORF47, encodes glycoprotein L. The preferential enhancement of genes involved in KSHV DNA replication is consistent with the effect of IFIT KD on KSHV DNA replication shown above. However, the increased expression of the majority of KSHV lytic genes suggested that IFITs may have general effects beyond inhibition of specific KSHV genes.

In order to determine whether IFITs have a generally inhibitory effect on KSHV replication, we set out to examine the effect of IFIT KD in the BCBL1 KSHV-infected primary effusion lymphoma (PEL) cell line. The TREx BCBL1-Rta cell line employed carries a doxycycline-inducible Rta gene, allowing robust KSHV lytic replication upon doxycycline treatment [32], kind gift of Jae Jung.

TRExBCBL1-Rta cells were treated with doxycycline and cells were harvested at serial time points from 0–48 hrs at 12 hr intervals. IFIT1 and IFIT3 protein were not detectable (S6A Fig, S6B Fig) by Western blotting although KSHV ORF57 was strongly induced (S6C Fig). IFIT expression in iSLK Bac16 cells was easily detectable under the same conditions. These results indicated that the innate immune response in TRExBCBL1-Rta is different from that in iSLK/Bac16, consistent with prior reports that IFIT expression is nonfunctional in a large percentage of cancer cell lines and primary cancer cells [33, 34]. Regardless, we asked whether KD of IFIT mRNAs could affect KSHV gene expression even though IFIT protein expression was undetectable by immunoblotting. Lentiviruses containing shIFIT1 were constructed and tested by infecting induced iSLK/Bac16. Three clones of shIFIT1s (258, 316 and 581) achieved efficient knockdown of IFIT1 in iSLK/Bac16 without cellular toxicity (S6D Fig). These three lentiviral preparations were combined and used to infect TRExBCBL1-Rta at an MOI of 15. After infection by lentivirus, cells were either treated or mock-treated with doxycycline and harvested at 48hr post induction. Flow sorting was performed to confirm transduction by GFP-expressing lentivirus. Lysates from control or IFIT1 shRNA transduced TREX BCBL1-Rta cells showed no differences in ORF57 (early) or K8.1 (late) lytic protein expression (S6G Fig and S6H Fig). These results indicate that IFITs are not expressed and do not restrict KSHV lytic replication in BCBL1 cells.

### IFIT depletion downregulates the cellular type 1 interferon pathway

**Fig 5:**
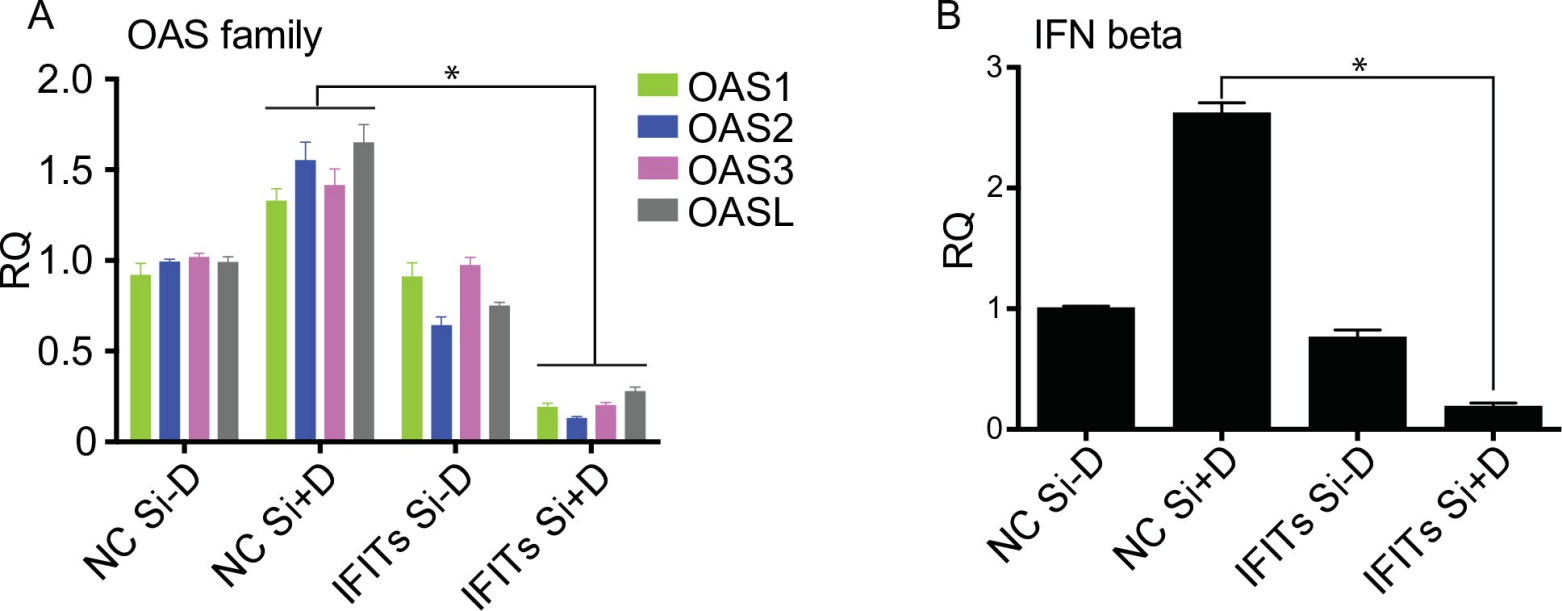
Effects of IFITs knockdown on expression of OAS family and IFN β. iSLK cells were depleted of IFIT1, IFIT2 and IFIT3 (IFITs) with siRNAs or transfected with negative control siRNA (NC Si). Cells were either untreated or treated with doxycycline induced to permit lytic KSHV replication. RNA was harvested at 48 h from each sample and qPCR was performed with primers specific for OAS1, OAS2, OAS3, OASL (A) or INF β (B). Expression of each mRNA (relative quantity, RQ) was normalized to the level of expression in uninduced control cells (NC Si-D). Each experiment was performed in biological triplicate and PCR was performed as technical triplicates. Error bars show SEM. *P < 0.0002.

IFIT depletion enhanced KSHV DNA replication and upregulated viral mRNA expression, contributing to increased virion production. We also wished to determine the effects of IFIT KD on cellular gene expression. There were 99 cellular genes whose transcript abundance decreased by 50% or more upon IFIT KD when KSHV lytic replication was induced. Analysis of this gene set by GO Enrichment Analysis (http://geneontology.org/page/go-enrichment-analysis) [35–37] showed high enrichment in genes assigned to the type 1 interferon pathway. 11 of the 99 genes whose transcript abundance decreased on IFIT KD were assigned to this pathway (Table 1). This represents a 35 fold enrichment over expected (p value 6.94 X 10^−14^). Among these ISGs, the OAS family (OAS1, OAS2, OAS3, OASL) was most significantly enriched. We performed qPCR for these OAS genes to validate and confirm the RNA Seq data (Fig 5A). All OAS genes were significantly down regulated (p<0.0002) to less than 17% after IFITs were depleted compared to mock depletion, suggesting that IFITs may affect KSHV replication through the OAS-RNase L pathway. Since several other ISGs belonging to the type 1 interferon pathway were also downregulated by IFIT KD, we performed qPCR to measure the expression of the most upstream regulator, IFN β. Lytic replication of KSHV induced IFN β expression in the control transfection (Fig 5B). IFN β expression was downregulated significantly (p<0.0001) to 7% in IFIT KD cells compared to the control (Fig 5B). Therefore, IFITs appear to enhance IFN β production and downstream ISG expression during KSHV lytic replication, and depletion of IFITs results in a blunted type 1 interferon response.

**Table 1:** Cellular genes belonging to type 1 interferon pathway are downregulated when IFITs are depleted.

Mapped ID	gene name/ gene symbol	Panther protein class	Log2 Ratio of difference*
OAS2	2'-5'-oligoadenylate synthase 2;OAS2	defense/immunity protein(PC00090);nucleic acid binding(PC00171);nucleotidyltransferase(PC00174)	-2.591378
OAS3	2'-5'-oligoadenylate synthase 3;OAS3	defense/immunity protein(PC00090);nucleic acid binding(PC00171);nucleotidyltransferase(PC00174)	-1.913599
OAS1	2'-5'-oligoadenylate synthase 1;OAS1	defense/immunity protein(PC00090);nucleic acid binding(PC00171);nucleotidyltransferase(PC00174)	-1.792586
XAF1	XIAP-associated factor 1;XAF1		-1.547982
RSAD2	Radical S-adenosyl methionine domain-containing protein 2;RSAD2		-1.441389
OASL	2'-5'-oligoadenylate synthase-like protein;OASL	defense/immunity protein(PC00090);nucleic acid binding(PC00171);nucleotidyltransferase(PC00174)	-1.346955
IFI35	Interferon-induced 35 kDa protein;IFI35	transcription cofactor(PC00217)	-1.29726
IFI6	Interferon alpha-inducible protein 6;IFI6		-1.244741
MX2	Interferon-induced GTP-binding protein Mx2;MX2	hydrolase(PC00121);microtubule family cytoskeletal protein(PC00157);small GTPase(PC00208)	-1.216792
IFI27	Interferon alpha-inducible protein 27, mitochondrial;IFI27		-1.065228
HLA-B	HLA class I histocompatibility antigen, B-73 alpha chain;HLA-B		-1.078583

* Log_2_ ratio of IFITs Si + Dox vs NC sI + Dox

### IFIT KD leads to decreased OAS function, RNase L activity and RNA accumulation

**Fig 6:**
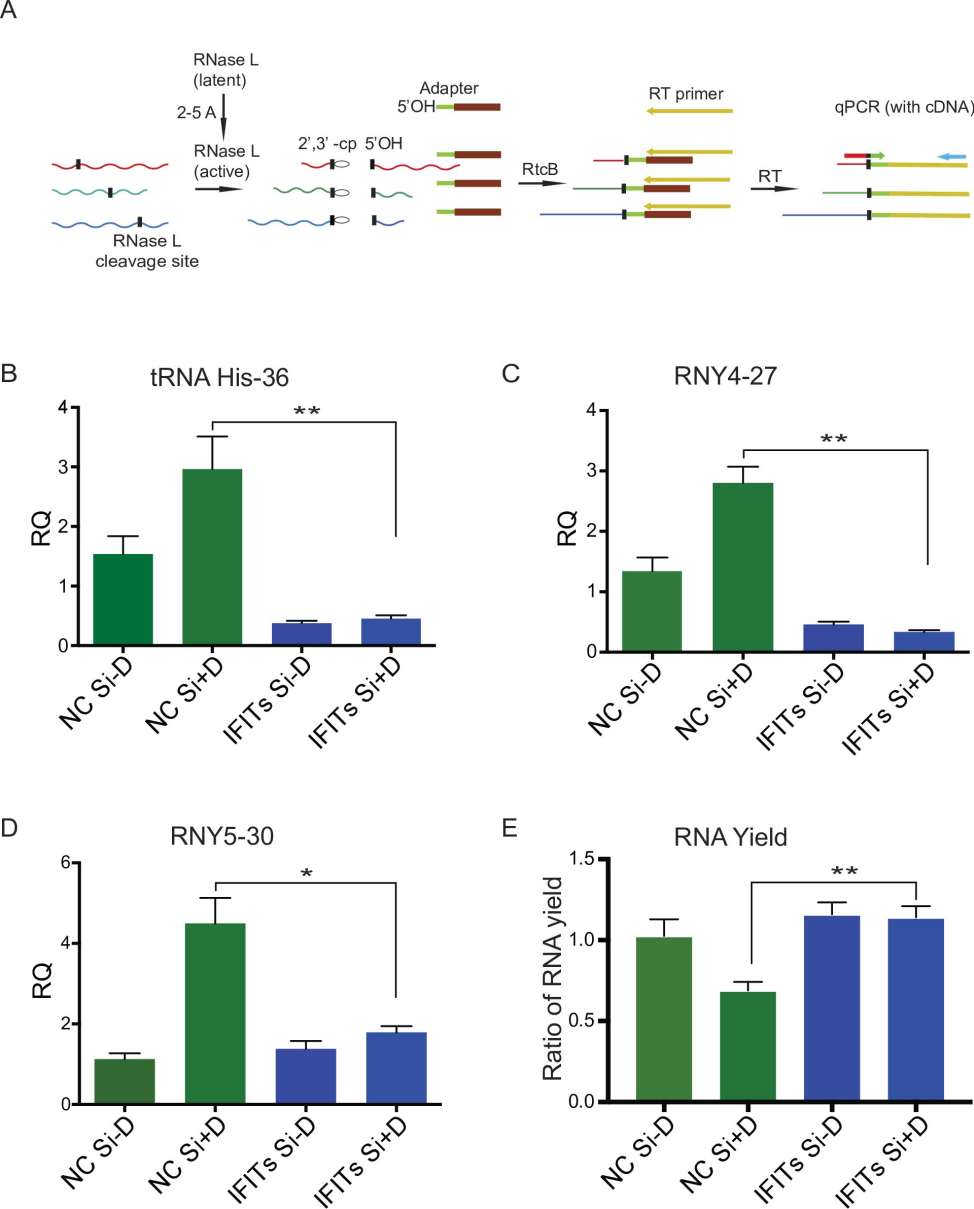
Activation of RNase L in cells depleted of IFIT2, IFIT1 and IFIT3. iSLK cells were depleted of IFIT1, IFIT2 and IFIT3 (IFITs) with siRNAs or mock depleted with negative control siRNA (NC Si). Cells were induced to permit lytic KSHV replication by treatment with doxycycline or mock-induced. RNA was harvested at 48 hr from each sample. U6 was used for normalization. (A) Diagram of site-specific qPCR for detection of RNase L site-specific cleavage. RNAs cleaved by RNase L and containing a 2′,3′-cyclic phosphate (waved lines, black vertical bar) were ligated using RtcB to an RNA-DNA adaptor (green-brown) containing a 5’OH RNA (green). The EDTA-quenched ligation reaction was used as a template for reverse transcription with Multiscribe RT. Reverse transcription was carried out using a primer with a 3′-end complementary to the adaptor and a 5′-overhang that serves as a universal priming site (yellow). SYBR-green based qPCR was conducted using a universal reverse primer (blue) that binds to the cDNA overhang and cleavage site-specific forward primers designed for each RNA target complementary to the RtcB ligation junction (red-green). (B-D) Quantitation of RNase L activity. Three specific RNase L cleavage site in His-tRNA and Y RNA were measured. (E) Total RNA yield from cells induced to permit lytic replication and transfected with either IFITs siRNA or NC siRNA. Results are shown as the ratio of each RNA amount to the yield from uninduced, NC transfected cells. Error bars show SEM of qPCR or ratio of RNA yield from three biological replicates; *P < 0.05, **P < 0.01.

The 2’,5’-oligoadenylate (2-5A) synthetase (OAS)-RNase L system is an interferon-induced antiviral pathway. Induction of OAS proteins by IFN and viral replication leads to synthesis of 2’5’ oligoadenylates which activate RNase L. OASs (OAS1, OAS2, OAS3) synthesize 2’-5’ oligoadenylates and activate RNase L leading to degradation of viral and cellular RNAs, thereby restricting viral infections [38]. Activated RNase L preferentially cleaves target RNAs produced by viruses as well as several cellular RNAs at specific sites [39, 40]. To confirm that the OAS expression that was inhibited by IFIT KD was functionally relevant, we compared RNase L activity in IFIT depleted and mock-depleted cells. Donovan et al. have demonstrated that RNA cleavage by activated RNase L can be quantitatively measured by RtcB-ligase assisted qPCR [40]. In this assay, RtcB ligase, which is capable of ligating 2’,3’-cyclic phosphates (generated by RNase L cleavage) to 5’OH RNAs, is used to ligate all such ends in the total cellular RNA pool to an RNA-DNA adapter with a 5’OH group. The ligated RNA is then reverse-transcribed and the cDNA is analyzed by qPCR. By using forward primers complementary to specific individual cleavage sites in known RNase L targets, measurement of the cleavage at each such site is achieved, serving as a quantitation of RNase L activity (Fig 6A). In order to serve as an internal control, U6 RNA, which has a naturally occurring cyclic 2’3’ phosphate, was also analyzed and used for normalization. We prepared and purified recombinant RtcB (S7 Fig) and then performed a ligation-PCR assay to measure the effect of OAS downregulation on RNase L activity. The RtcB analysis demonstrated that upon IFIT KD, RNase L cleavage decreased significantly at site 36 in tRNA-His, site 27 in non-protein-coding RNA RNY4 and site 30 in non-protein-coding RNA RNY5. The differences between RNase L directed cleavage in the presence and absence of IFITs were statistically significant as shown in Fig 6B–6D. Consistent with lower cleavage activity of RNase L, total RNA yield increased significantly upon IFITs KD compared to the control (Fig 6E). Thus, IFITs may inhibit KSHV replication through the OAS-RNase L pathway.

### IFIT1 and IFIT3 bind KSHV mRNAs

**Fig 7:**
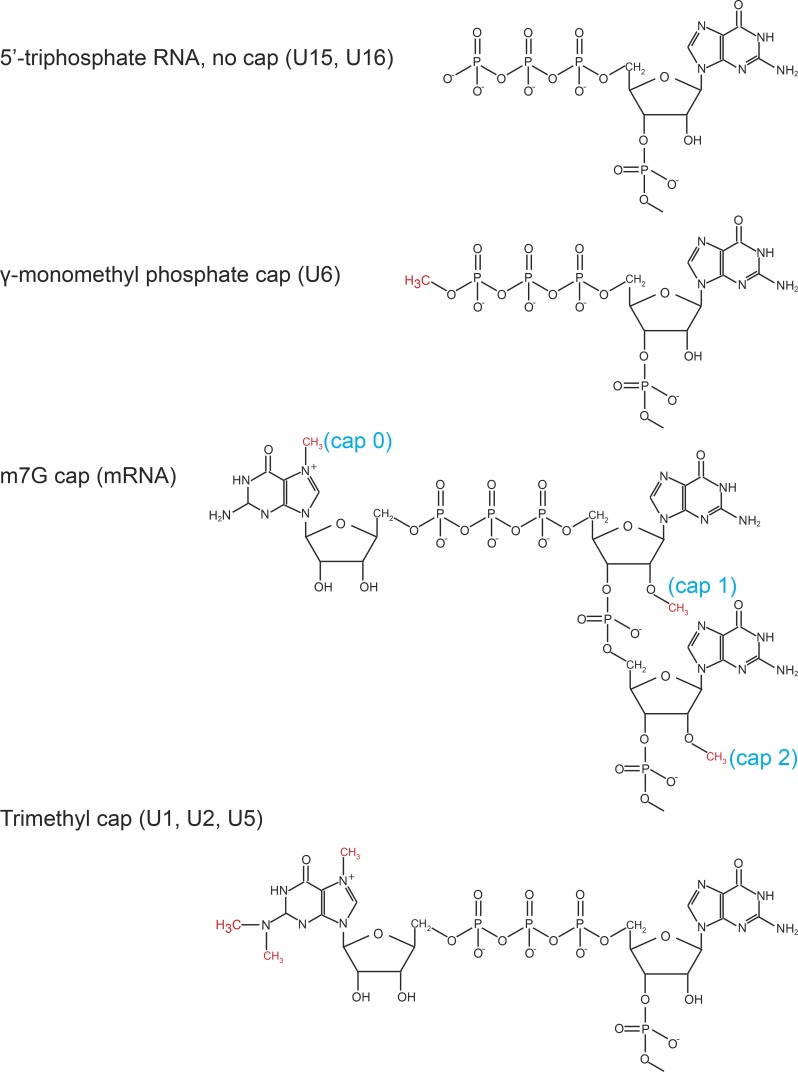
Structures of RNA 5’ caps. Structures of RNAs with 5-triphosphate (no cap), γ-monomethyl phosphate cap, m7G caps (cap 0, cap1, cap2) and trimethyl cap. Methyl groups are shown in red and the various types of caps are labeled in blue. Based on structures described in [66].

**Fig 8:**
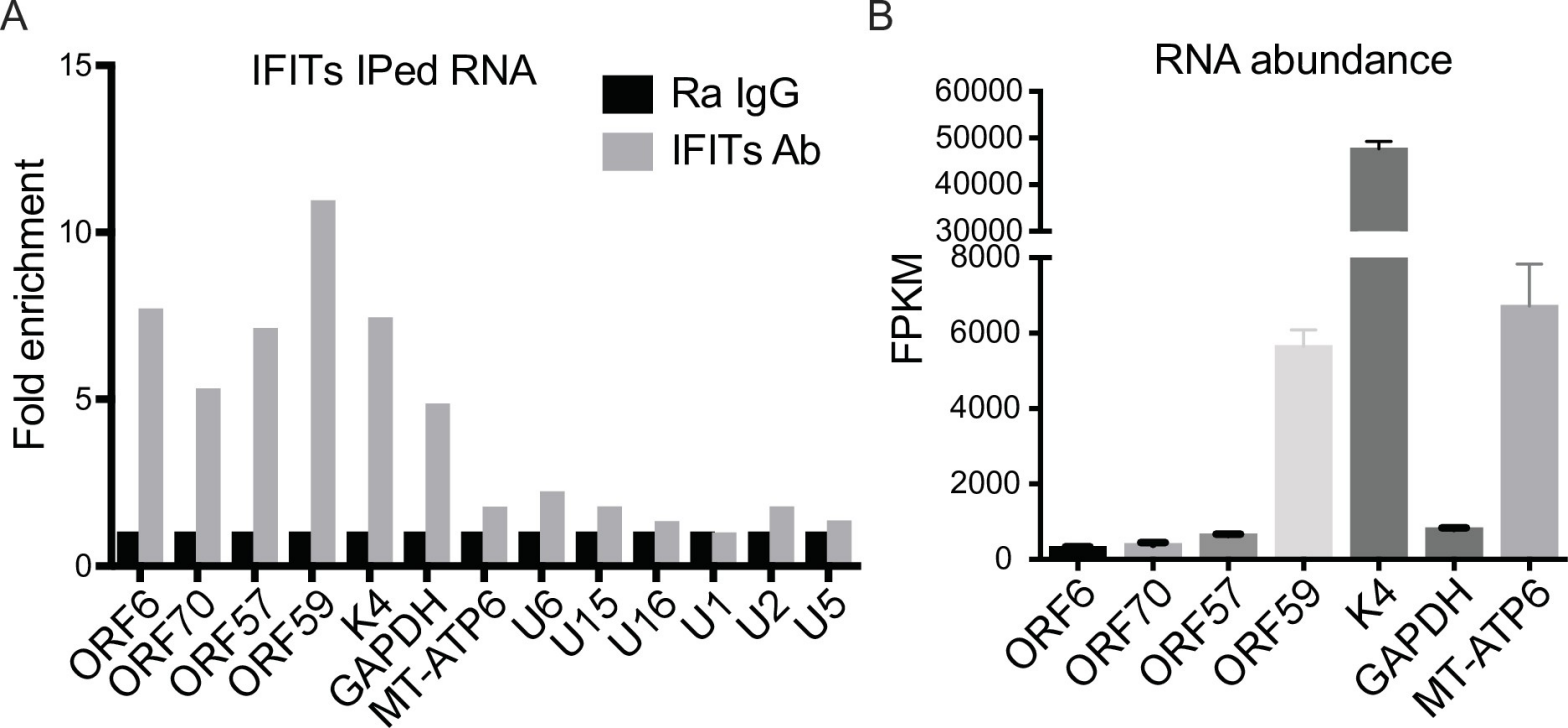
IFIT1, IFIT2 and IFIT3 immunoprecipitate KSHV mRNAs. A. Quantitation of RNAs immunoprecipated (IPed) with IFITs. iSLK cells were treated with 1 μg/ml doxycycline to induce KSHV replication and cells were harvested at 48 hr for immunoprecipitation with IFIT1 and IFIT3 antibodies. qPCR was performed to measure IFIT1 and IFIT3 immunoprecipitated viral and cellular RNA. Fold enrichment over control immunoprecipitations are shown. B. Transcript abundance of selected KSHV genes highly upregulated by IFIT KD. Fragments per kilobase of transcript per million mapped reads (FPKM) from RNA sequencing of viral and control cellular genes are shown.

IFIT1 has been shown to preferentially recognize certain types of capped RNA [12, 41]. Recently published data indicate that IFIT3 stabilizes IFIT1 and increases its affinity for cap0 mRNAs (See Fig 7 for a diagram of the various types of RNA 5’ caps) [41–43]. However, IFIT1 is also capable of binding cap1 mRNAs (Fig 7), albeit at lower affinities [41]. It was therefore possible that IFIT1 might recognize and inhibit translation or stability of KSHV mRNAs directly in addition to indirect effects mediated via other ISGs as shown with OASs. We performed immunoprecipitation experiments to determine if IFIT1 bound viral mRNA specifically or preferentially. iSLK cells were treated with doxycycline to induce KSHV replication, lysates were harvested at 48 hr post induction and immunoprecipitated with IFIT1 and IFIT3 antibodies. Immunoprecipitated RNAs were isolated and measured by qPCR. As shown in Fig 8A, viral RNAs were enriched 5~11-fold in the immunoprecipitation using IFIT1 and IFIT3 antibodies compared to control IPs. The cellular GAPDH mRNA was enriched 4.8-fold. It should be noted that the degree of binding of individual RNAs to IFITs was not related to their overall abundance. For example, ORF6, which was present at 330 FKPM was enriched similarly to K4, which was highly abundant at 50,000 FKPM (Fig 8B). Uncapped MT-ADP6 RNA, a cellular mitochondrial transcript [44], as well as uncapped snoRNAs U15 [45] and U16 [46, 47] were enriched only 1.7-fold (Fig 8A). U6 RNA, which has a gamma-monomethyl phosphate cap [48], was enriched in only 2.2-fold in the immunoprecipitates (Fig 8A). U1, U2 and U5 have a trimethylation cap [49], and they were similarly enriched less than 1.8-fold (Fig 8A). All these snoRNA are expressed at more than 10,000 copies per cell which is a much higher abundance compared to cellular genes [50, 51]. Therefore, IFIT1 and IFIT3, while preferentially recognizing cap0 structures, are also capable of binding to both viral and cellular capped mRNA (cap1 or cap2). However, their ability to bind uncapped, monomethyl capped, or trimethyl capped RNAs appears to be extremely limited.

## Discussion

In this study we examined the effects of cellular IFIT1, IFIT2 and IFIT3 on KSHV lytic replication. Heretofore, IFIT proteins have been primarily implicated in antiviral responses against RNA viruses [18, 19]. IFIT1, IFIT2 and IFIT3 form multimeric complexes that initially were shown to bind 5’ tri-phosphate RNAs [25]. Such RNAs are produced by several negative strand RNA viruses such as Rift Valley virus, vesicular stomatitis virus and influenza virus against which IFITs exhibit antiviral activity [19]. However, IFITs were subsequently shown to also preferentially bind cap0 mRNAs which lack 2’O-methylation at the first and second transcribed ribonucleotides (as seen in cap1 and cap2 mRNAs, Fig 7). Many viruses that replicate in the cytoplasm, including flaviviridae, poxviridae and coronaviridae, have evolved enzymes to independently perform 2’O-methylation of their mRNAs. Mutants of these viruses that have lost 2’O methyltransferase activity exhibit increased susceptibility to IFIT dependent immune responses, suggesting that IFITs allow discrimination between self and non-self RNAs [12, 52]. Although IFIT induction by RNA viruses is common, herpesviruses may also induce IFIT gene expression, by direct or indirect mechanisms. Indeed, IFIT2 and IFIT3 were identified as hCMV induced genes (cigs) over twenty years ago by the use of differential display [27]. HSV infection also leads to IFIT induction, albeit less strongly than CMV infection [27]. It was therefore of interest to determine whether a gammaherpesvirus such as KSHV could induce IFIT gene expression. KSHV, similar to other herpesviruses which undergo lytic replication in the nucleus, are presumed to have cap structures similar if not identical to host mRNAs [24]. Nevertheless, IFIT1 has also been shown to exert inhibitory effects on translation independent of mRNA sequestration by interacting directly with eIF3 [18], also raising the question of whether IFITs could establish an antiviral state that would inhibit KSHV virion production.

We first established that KSHV reactivation and lytic replication results in IFIT induction. While all three IFIT mRNAs were measurably induced upon KSHV lytic replication, we were only able to detect increased expression of IFT1 and IFIT3 proteins. While the IFIT2 antibodies we employed were able to detect exogenously overexpressed IFIT2, we did not detect IFIT2 protein expression by either immunoblotting or immunofluorescence microscopy. This may be due to minimal IFIT2 protein induction as a consequence of KSHV replication but our finding that IFIT2 depletion enhanced KSHV production suggests that functional IFIT2 is present and the failure to detect IFIT2 protein is likely due to inadequately sensitive IFIT2 antibodies. Nevertheless, it is clear that although KSHV lytic reactivation occurs from the nucleus, PAMP exposure sufficient to engage PRRs and induce ISG expression occurs. Future studies to examine the nature of the non-self signatures, whether DNA, RNA or protein, that evoke the innate immune response to reactivating herpesviruses, and whether the PRRs that recognize them are nuclear and/or cytoplasmic, will be very informative. Both IFIT1 and IFIT3 proteins that we detected by IF studies were localized to the cytoplasm in KSHV infected cells. Although virtually all studies have focused on the interaction of IFITs with cytoplasmic RNAs, it has been suggested that IFIT1 may also have transcriptional activating functions [53].

We examined the potential role of IFITs as KSHV inhibitory proteins by knocking down IFITs and then inducing KSHV lytic replication. As expected, IFIT expression was minimal in the absence of KSHV replication, and expression of IFITs was also severely curtailed after siRNA treatment. Blocking IFIT production resulted in a 25–30 fold increase in infectious KSHV virion production. Consistent with these findings, IFIT KD also led to increased lytic KSHV mRNA accumulation. Importantly, the increase in mRNA abundance, while not completely equal amongst all KSHV mRNA, was nevertheless broad, with over 65% of lytic mRNAs increasing in abundance. However, these findings pose a difficulty in interpretation due to the fact that herpesvirus late gene transcription, including that of KSHV, is dependent on DNA replication [54]. Since the most highly IFIT restricted KSHV mRNAs encode proteins that are either essential or important for KSHV lytic DNA replication, the broad inhibitory effect on KSHV mRNAs may be partly indirect, with late gene repression by IFITs due to the inhibitory effect of IFITs on viral DNA replication. We confirmed that IFITs do have an inhibitory effect on DNA replication by directly measuring KSHV DNA abundance in the presence and absence of IFITs.

KSHV is thought to most likely enter the human host by oral epithelial cell infection [55] and has been demonstrated to infect a variety of human epithelial cells including oral keratinocytes as well as epithelial cell lines [56, 57]. These experiments were carried out in the iSLK/Bac16 cell line, an epithelial cell line that supports efficient KSHV lytic replication and has served as a model for KSHV infection and reactivation from latency [58]. We also examined the potential role of IFITs in KSHV reactivation from latent infection in a PEL cell line, BCBL1 [59]. However, cells from BCBL1 did not express detectable IFITs and it was therefore not possible to determine whether IFITs may play a role in restricting KSHV replication in B lymphocytes. As loss of ISG expression is not uncommon in many human tumors, these findings do not rule out the possibility of IFITs playing a physiological role in regulating KSHV replication in B lymphocytes in vivo.

These data also raised the possibility that IFITs exerted at least some of their antiviral function by other indirect mechanisms that did not depend on specific targeting of KSHV mRNAs, especially since there are no known differences in cap structures between herpesvirus mRNAs and host cellular mRNAs [24]. Our analysis of the cellular transcriptome suggested an effect of IFITs on the type I interferon pathway, as several ISGs, including several known to be important for establishing an antiviral state, decreased upon IFIT KD in comparison to infected cells in which IFITs were not depleted. These findings do not differentiate between transcriptional or post-transcriptional effects of IFITs in enhancing ISG expression. However, the fact that interferon mRNA levels were decreased in the absence of IFITs suggests that the simplest model for a positive feedback loop maintained by IFITs might be an effect on type I IFN transcription or RNA stability. A recent report implicated IFIT1 in nuclear regulation of transcription, both acting to negatively regulate the inflammatory response as well as enhancing IFN β1 transcription [53]. The IFN β 1 response to pathogens was also blunted in IFIT1-depleted cells in this study. Our data support a model in which IFITs maintain an antiviral state by promoting enhanced IFN and ISG production.

OAS proteins are established components of the innate immune response to viruses. OASs synthesize 2’-5’ oligoadenylates and activate RNase L leading to degradation of viral and cellular RNAs and thereby block viral infections as well as amplification of IFN α/β by RNase L-generated small RNAs [38]. Because OAS mRNAs were the most highly downregulated upon IFIT KD, we examined whether this correlated with a functional decrease in potential antiviral activity. By using the RtcB-ligase assay, which measures the production of cyclic 3’ phosphate moieties at specific RNase L cleavage sites, we were able to determine that ISG KD does lead to a functional decrease in RNase L activity. The observed decrease in RNase L activity is consistent with the generalized decrease in KSHV lytic mRNA abundance and in total cellular RNA.

We also examined the ability of IFITs to bind several types of capped RNA. By direct immunoprecipitation of IFIT proteins, we found that cap1/2 host cell mRNAs and KSHV mRNAs were widely represented in IFIT immunoprecipitates. Despite the fact that IFIT complexes were originally isolated by using triphosphate uncapped RNAs as bait, we found very little representation of naturally uncapped RNAs such as certain mitochondrial or snoRNAs [44–47, 60] in IFIT immunoprecipitates. Whether interactions of IFITs with cap1/2 mRNAs exert negative effects on their stability or translation or if such interactions could even have positive effects on target mRNA remains to be determined. Although the enhanced affinity of IFIT1 complexes for cap0 mRNAs has been adduced as evidence of a PAMP recognition by IFITs that allows them to distinguish between self and non-self, the fact remains that the majority of viral mRNAs are 2’-O-methylated. In addition, some viruses, such as parainfluenza virus, whose mRNAs are 2’-O-methylated, are nevertheless inhibited by IFITs [61]. Our findings demonstrating that IFITs exert antiviral effects on KSHV, a herpesvirus, which has neither genomic RNAs nor atypically capped mRNAs, provide further evidence that IFITs may have antiviral effects beyond direct sequestration of mRNA. Although IFIT complexes do not appear to be highly enriched for specific transcripts, we cannot rule out the possibility that sequestration by IFITs may have varying effects on different targets, affording a degree of specificity. Variation in the effects of IFIT binding to different targets could arise from intrinsic differences in translatability or stability of individual target mRNAs, especially lytic herpes virus transcripts, which are primarily intronless [62, 63].

In summary we have shown that IFITs exert an antiviral effect on a herpesvirus which does not express any of the putative pathogen associated RNA signatures expressed by RNA viruses. In addition, in infected cells, IFITs do associate with canonically capped viral and cellular mRNAs that are not known to be possess cap0 structures. Further, IFIT depletion led to decreases in IFNβ as well as several other antiviral effectors of the interferon pathway, suggesting that IFITs may possess broad antiviral effects by virtue of their ability to amplify the interferon response. Finally, by virtue of the IFITs ability to interact with canonically capped cellular and viral transcripts, they may also affect both host cell and viral gene expression by direct effects on mRNA.

## Materials and methods

### Cells and plasmids

293T cells (kind gift of Lori Frappier, University of Toronto) were grown at 37°C in Dulbecco’s modified Eagle’s medium (DMEM) supplemented with 10% fetal bovine serum (FBS) and glutamine. iSLK cells [58] (gift of Don Ganem, UCSF) were maintained in DMEM containing 10% charcoal stripped FBS (Sigma) and 1% glutamine with 250μg/ml G-418 and 1μg/ml puromycin. iSLK cells were infected with WT KSHV derived from bacmid BAC16, expressing eGFP and hygromycin resistance [28]. Bac16 KSHV infected iSLK cells were maintained in 1.2 mg/ml hygromycin, 250μg/ml G-418 and 1μg/ml puromycin. TRExBCBL1-Rta (kindly provided by Prof. J. Jung) were cultured in RPMI 1640 10% Tet System approved FBS (Clontech) and 1% glutamine with 50μg/ml hygromycin.

### IFIT1, IFIT2 and IFIT3 knockdown

IFIT1 (L-019616-00-0005), IFIT2 (L-012582-02-0005), IFIT3 (L-017691-00-0005) and negative control On-target plus Smart Pool siRNAs (D-001810-03) were purchased from Thermo Scientific. Each siRNA was transfected into iSLK cells using Lipofectamine RNAiMAX (Invitrogen) according to the manufacturer’s protocol. For KD of all three IFITs, each siRNA was used at 10 nM final concentration and NC siRNA was used at 30 nM. For individual IFIT KD, each siRNA or NC siRNA was used at 10 nM. RT-qPCR or immunoblotting was performed to verify knockdown of the relevant protein.

### Preparation of shRNA lentiviruses and lentivirus infection

IFIT1 GIPZ shRNA clones (RHS4531-EG3434) were purchased from Dharmacon. Lentiviruses were prepared by transient transfection of 293T cells with a three-plasmid system (a GIPZ plasmid expressing shRNA against cellular IFIT1; pMD2.G [envelope plasmid expressing vesicular stomatitis virus glycoprotein]; and psPAX2 [packaging plasmid]). Viral supernatant was harvested at 48hr post transfection with 0.45μm syringe filter. Lentivirus were concentrated at 10000g in 10% sucrose buffer for 3.5hr as described [64] and immediately used to infect iSLK/Bac16 or TREx BCBL1-Rta cells. iSLK/Bac16 cells were infected with each lentivirus at an MOI of 15 and then induced to permit KSHV lytic replication. Cells were harvested 48hr post-induction and Western blotting of IFIT1 was performed. Lentiviruses containing three independent shRNAs were used to infect TRExBCBL1-Rta cells at an MOI of 15. For shRNA knockdown experiments, TRExBCBL1-Rta cells were infected twice with concentrated lentiviruses within two days. 2 days after the second infection, cells were seeded at 1 × 10^6^/ml and induced with 1μg/ml doxycycline. Cells were harvested or sorted by flow cytometry at 48 post induction.

### RNA isolation and analysis

Total cellular RNA was isolated from washed cell pellets using Qiazol and Qiagen miRNeasy columns according to the manufacturer’s protocols. mRNA was purified from 6 μg cellular RNA using Qiagen Oligotex mRNA Minikit (Qiagen). cDNA libraries were prepared using the ABI high Capacity cDNA Reverse Transcription Kit with RNase inhibitor (Applied Biosystems). Real-time quantitative PCR (qPCR) was performed with SYBR green PCR Master Mix (Applied Biosystems) according to the manufacturer’s protocol. Each sample was analyzed in triplicate with gene specific primers and β-actin was used as the endogenous control. The gene-specific primers were as follows:

IFIT1 Q1F: 5’-ggaatacacaacctactagcc-3’;

IFIT1 Q1R: 5’-ccaggtcaccagactcctca-3’;

IFIT2 Q1F: 5’-gggaaactatgcctgggtc-3’;

IFIT2 Q1R: 5’-ccttcgctctttcattttggtttc-3’;

IFIT3 Q1F: 5’-tgaggaagggtggacacaactgaa-3’;

IFIT3 Q1R: 5’-aggagaattctgggttgttgggct-3’

OAS1 Q1F: 5’-gcgccccaccaagctcaaga-3’

OAS1 Q1R: 5’-gctccctcgctcccaagcat-3’

OAS2 Q1F: acccgaacagttccccctggt-3’

OAS2 Q1R: 5’-acaagggtaccatcggagttgcc-3’

OAS3 Q1F: 5’-tgctgccagcctttgacgcc-3’

OAS3 Q1R: 5’-tcgcccgcattgctgtagctg-3’

OASL Q1F: 5’-gcggagcccatcacggtcac-3’

OASL Q1R: 5’-agcaccaccgcaggccttga-3’

ORF6 Q1F: 5’-ctgccataggagggatgtttg-3’

ORF6 Q1R: 5’-ccatgagcattgctctggct-3’

ORF47 Q1F: 5’-agcctctaccctgccgttgttct-3’;

ORF47 Q1R 5’-acgaccgcgactaaaaatgacct-3’;

ORF54 Q1F: 5’-gtagccgcatatgccagattgtg-3’

ORF54 Q1R: 5’-ttttgaagcccttgaggatgtgtc-3’

ORF56 Q1F: 5’-cacagattcccgtcaatacaaa-3’;

ORF56 Q1R, 5’-gtatcttcagtaggcggcagag-3’;

ORF57 Q1-5: 5’-gcagaacaacacggggcgga-3’

ORF57 Q2-3: 5’-gtcgtcgaagcgggggctct-3’

ORF70 Q1F: 5’-gactatacaggccaggggtttgac-3’

ORF70 Q1R: 5’-ggcgggttccacgcacac-3’

K4 Q1F: 5’-gtttgcaatctggggacacg-3’

K4 Q1R: 5’-tggtaaccgagacagcacttg-3’

β-actin Q1F: 5’-tcaagatcattgctcctcctgag-3’

β-actin Q1R: 5’-acatctgctggaaggtggaca-3’

### High-throughput deep sequencing of RNA and Bioinformatic analysis

High-throughput deep sequencing of RNA was performed as previously described [29] with some modifications. Briefly, iSLK cells were transfected with a mixture of 10 nM final concentration of each IFIT siRNA or negative control siRNA (30nM final concentration) and were treated with 1 μg/ml doxycycline after 6 hrs. Cells were harvested at 48 hr post induction for RNA isolation. RNA samples from iSLK cells were prepared using Qiagen miRNeasy kits according to the manufacturer’s protocols. cDNA libraries were prepared from poly(A) RNA and were sequenced on a HiSeq2000 instrument with 50 cycle single end reads. Sequenced reads obtained from Bac16 KSHV-infected iSLK cells were aligned to the KSHV Bac16 (GenBank accession no. GQ994935.1) and Hg19. Differential gene expression was measured using USeq’s Defined Region Differential Seq application as described previously [29].

### Immunoblotting analysis

Protein samples were analyzed by sodium dodecyl sulfate polyacrylamide gel electrophoresis (SDS-PAGE) and immunoblotted with rabbit polyclonal anti-IFIT1 antibody (PA3-848, ThermoScience), rabbit polyclonal anti-ORF57 antibody [65], mouse anti-K8.1 monoclonal antibody (kind gift from Bala Chandran), anti-α tubulin polyclonal antibody (Sigma) or mouse anti-actin monoclonal antibody (MA5-15739, Thermo Science) and horseradish peroxidase-conjugated anti-rabbit secondary antibody (NA934V, GE Healthcare) or anti-mouse secondary antibody (NA931V, GE Healthcare), followed by visualization with a Prometheus ProSignal Peroxide Kit (Genesee Scientific). Image capture and densitometry quantitation of IFIT1 and IFIT3 proteins was performed with a BioRad GelDoc system and Image Lab software (V5.1). Final depletion was calculated as percentage of IFIT KD from NC control and normalized to tubulin.

### Immunofluorescence microscopy

iSLK cells were grown on glass coverslips plated at 120,000 cells per well in six-well dishes and treated with 1 μg/ml doxycycline to induce virus lytic replication. Cells were fixed at 48hr, 72hr and 96hr post induction, washed with 1x PBS, fixed and permeabilized with PBS containing 4% paraformaldehyde and 0.2% Triton X-100 for 15–20 minutes at room temperature, and then washed two times with 1x PBS followed by incubation with blocking buffer (20% goat serum in PBS) for 30 mins at room temperature. Cells were incubated with anti-IFIT3 polyclonal antibody (Thermo Science) or anti-IFIT1 polyclonal antibody (Thermo Science) at a dilution of 1:500, at 37°C for 1 hour. The slides were washed three times with 1x PBS and incubated with Alexa Fluor 594 goat anti-rabbit IgG (A11072, Invitrogen) for 1 hour at 37°C (in the dark). Nuclear staining was performed with 4',6- diamidino-2-phenylindole (DAPI) (Invitrogen). Images were collected and analyzed with a Zeiss Imager M2 microscope system. To determine cells staining positive for IFIT3, more than 6 fields in which each field included more than 2000 cells (totally at least 12000 cells) were counted with a 20x objective. Statistical testing for comparison of proportions and p values was performed using MedCalc software, which uses the "N-1" Chi-squared test (MedCalc software, Ostend Belgium)

### Induction of lytic gene expression, virus replication and quantification of infectious virus release and virus replication

To induce KSHV lytic gene expression or virus replication, iSLK cells were treated with 1 μg/ml doxycycline. Where KD of IFITs was performed, siRNA transfections were performed 6 hours prior to induction. Cells were harvested at 48 hr post induction for RNA preparation. At 48 hrs post-induction, cell viability was greater than 94% by vital dye staining. For virus production, supernatants of the cells were harvested 5 days post induction, cleared by centrifugation twice, and filtered through a 0.80 μm pore size cellulose acetate filter. Serial dilutions of supernatants were used to infect 293T cells. 48 hours after infection, flow cytometry was performed to measure the number of GFP positive cells, each representing a cell infected by a GFP expressing KSHV virion [29]. Each infection was done in triplicate and each infected cell sample was assayed by flow cytometry in technical triplicates. Based on the dilution factor, infectious virus titers in the iSLK cell supernatant were calculated. Pellets of the cells from which supernatant was harvested were processed for DNA isolation using Qiagen DNeasy Blood and Tissue kit. 50 ng of each DNA were used for qPCR using primers specific for ORF59 (see above) and SYBR green PCR MasterMix (ABI).

### RtcB enzyme preparation

RtcB expression plasmid (kind gift from Dr. Alexei V. Korennykh), contains the RtcB gene from E. coli cloned into pGEX-6P and expressed with an N-terminal GST-tag. Protein isolation and purification were performed as previously described [40] with some modifications. Briefly, E. coli BL21-CodonPlus (DE3) carrying the RtcB expression plasmid was grown at 37°C in LB medium containing 50 μg/ml ampicillin until the A600 reached 0.6 to 0.7. The culture was chilled down to 20°C and protein expression was induced by IPTG (0.25mM final concentration). Incubation was continued at 20°C for 16 hr with constant shaking. Cells were harvested by centrifugation and stored at −80°C. All subsequent procedures were performed at 4°C. The cell pellet was suspended in 50 ml buffer A (20 mM HEPES pH 7.5, 300 mM NaCl, 10% glycerol, 2 mM DTT, 0.1 mM EDTA, 1% Triton X-100) with proteinase inhibitor (0.3μg/ml aprotinin, 0.5μg/ml leupeptin and 0.7μg/ml pepstatin A), 10μg/ml DNase and 10μg/ml lysozyme. After mixing for 30 min, cells were sonicated and insoluble material was removed by centrifugation. The clarified lysate was added to a column with 10ml glutathione resin washed with buffer A. The column was rotated for 1h at 4°C to bind GST-RtcB to the resin. Washing was performed with buffer A and the resin was resuspended in low salt buffer A (100mM NaCl without Triton X-100). PreScission Protease (GE Healthcare) was added and the column was gently rotated overnight at 4°C. The cleaved RtcB was then eluted from the column in 10ml buffer A. Ion exchange (MonoQ) purification (S7 Fig) followed by S200 gel filtration (S7 Fig) was performed with unsalted buffer (20 mM HEPES pH 7.5), high salt buffer (20 mM HEPES pH 7.5, 1M NaCl) and buffer B (buffer A without 1% Triton X-100). Purified RtcB was eluted and diluted to 100μM in buffer B with 0.5% Triton X-100, aliquoted and stored at -80°C.

### RNase L activity assay by RtcB-ligase assisted qPCR

We measured several specific cleavage sites generated by RNase L to analyze its activity as described [40]. Briefly, RtcB ligase and RtcB ligation adaptor (5’-OH-GAUCGUCGGACTGTAGAACTCTGAAC-3’) were added to cellular RNA (500ng) to ligate 2′,3′-cyclic phosphate containing RNAs to the adapter. The underlined bases in the ligation primer were RNA and the remainder were DNA. EDTA-quenched ligation reaction (1μL) was used as a template for reverse transcription with Multiscribe reverse transcriptase (ThermoFisher) and RT primer (5′-TCCCTATCAGTGATAGAGAGTTCA GAGTTCTACAGTCCG-3′). SYBR-green based qPCR was conducted using a universal rev qPCR primer (5’-TCCCTATCAGTGATAGAGAG-3’) and cleavage site-specific forward primers designed for each RNA target: tRNA His-36 (5’-GTTAGTACTCTGCGTTGTGGA-3’), RNY4-27 (5’-GATGGTAGTGGGTTATCAGAT-3’) and RNY5-30 (5’-GTGTTGTGGGTTATTGTTAGA-3’). U6, which has a naturally occurring 2′,3′-cyclic phosphate and an RNase L independent cleavage site, was used as endogenous control. Primers for the U6 site were U6 Q1F: 5’-GCTTCGGCAGCACATATACTA-3’ and U6 Q1R: 5’-CGAATTTGCGTGTCATCCTTG-3’, qPCR was carried out for 60 cycles using 62°C annealing/extension for 1 min.

### Isolation of IFIT1 and IFIT3 bound RNAs

iSLK/Bac16 cells were treated with 1 μg/ml doxycycline to induce KSHV replication. Cells were harvested at 48 hr post induction for immunoprecipitation. Cells were lysed by freeze-thawing in hypotonic buffer containing 20mM HEPES, pH7.3, 2mM MgCl_2_, 10% glycerol, 0.2mM EGTA, 1mM DTT, 1x protease inhibitor cocktail (Sigma) and RNasin (Promega). All subsequent steps were performed at 4°C. Lysates were clarified by centrifugation and precleared with normal rabbit IgG (Bethyl) and protein A-agarose beads, followed by immunoprecipitation with anti-IFIT1 plus anti-IFIT3 Rabbit polyclonal antibody (ThermoScience), or normal rabbit IgG overnight, followed by incubation with protein A-agarose beads. The beads were washed four times in IP washing solution (500 mM NaCl, 0.25% NP-40, 0.25% Triton X-100, 0.5% CHAPS). Coimmunoprecipitated RNA was isolated from the immunoprecipitates using Qiazol with additional glycogen and Qiagen miRNeasy columns according to the manufacturer’s protocols, with an on-column DNase treatment (Qiagen). Immunoprecipitated viral and cellular gene mRNA was quantitated by Real-time Quantitative PCR (qPCR) with SYBR green PCR Master Mix (Applied Biosystems) according to the manufacturer’s protocol. Each sample was analyzed in triplicate with gene specific primers. The gene-specific primers were as follows:

GAPDH Q1F, 5’-agggtcatcatctctgccccctc-3’;

GAPDH Q1R, 5’-tgtggtcatgagtccttccacgat-3’

MT-ATP6 Q1F: 5’-gtatgagcgggcgcagtgatt-3’

MT-ATP6 Q1R: 5’-atggggataaggggtgtaggtgtg-3’

U1 Q1F: 5’-ccatgatcacgaaggtggttt-3’

U1 Q1R: 5’-atgcagtcgagtttcccacat-3’

U2 Q1F: 5’-ctcggccttttggctaagat-3’

U2 Q1R: 5’-cgttcctggaggtactgcaa-3’

U5 Q1F: 5’-ctctggtttctcttcagatcgc-3’

U5 Q1R: 5’-ccaaggcaaggctcaaaaaat-3’

U6 Q1F: 5’-gcttcggcagcacatatactaaaattgga-3’

U6 Q1R: 5’-ataggaacgcttcacgaatttgcg-3’

U15 Q1F: 5’-ggtcacgtcctgctcttggtc-3’

U15 Q1R: 5’-atgcctctaaatcgatcaataaat-3’

U16 Q1F: 5’-atgatgtcgtaatttgcgtctt-3’

U16 Q1R: 5’-ctcagtaagaattttcgtcaacc-3’

KSHV gene specific primers are listed above.

## Supporting information

S1 Fig GFP positivity of iSLK/Bac16. Images of GFP positive KSHV-infected iSLK cells were collected and analyzed with a Zeiss Observer D1 microscope system (left panel). Right panel shows phase contrast microscopy of cells in left panel.
(TIF)

S2 Fig IFIT1, IFIT2 and IFIT3 expression in uninduced iSLK/Bac16. iSLK/Bac16 cells untreated (-D) with doxycycline were harvested at 48hr or 72hr post induction as shown. Immunoblotting of lysates from the cells was performed with anti-IFIT1 and anti-IFIT3 antibodies to measure IFIT1 (A) and IFIT3 (B) protein expression. Lysate from induced iSLK/Bac16 (KSHV-infected) at 72hr was used as a positive control in the rightmost lane (+D). Tubulin is shown as a loading control. qPCR was performed to measure IFIT RNA expression in the samples from 48hr and 72hr post induction as shown (C).
(TIF)

S3 Fig IFIT1 and IFIT3 expression in doxycycline treated iSLK in the absence of KSHV infection. iSLK cells (without KSHV infection) were mock-treated (-D) or treated with doxycycline (+D). Cells were harvested at 48hr or 72hr post induction (pi.) as shown. Immunoblotting of lysates was performed with anti-IFIT1 and anti-IFIT3 antibodies to measure IFIT1 (A) and IFIT3 (B) protein expression. Lysate from doxycycline induced iSLK/Bac16 at 72hr was used as a positive control on right (72/+D). Tubulin is shown as a loading control.
(TIF)

S4 Fig Immunofluorescence staining of iSLK/Bac16 cells for IFIT1. Cells were fixed at 48hr post-induction of lytic replication (pi) as shown. Cells were then stained for IFIT1 (Red). Arrows indicate magnified cells which are shown at right in the panel. DAPI staining of nuclei is shown in blue.
(TIF)

S5 Fig Effect of IFIT depletion on infectious virion production. Virion titration 2.KSHV-infected iSLK cells were transfected with either control siRNA (NC Si) or a mixture of IFIT1, IFIT2 and IFIT3-specific siRNA (IFITs Si) and KSHV replication was induced by treatment with doxycycline. Supernatants from induced cells were used to infect 293T cells. Virus passage was quantitated by flow cytometry of GFP-positive 293T cells. Each transfection/induction was performed in triplicate and three replicate infections were performed with each supernatant. Error bars show SEM of titration from triplicate samples.
(TIF)

S6 Fig IFIT1 and IFIT3 expression in doxycycline treated TRExBCBL1-Rta cells. TRExBCBL1-Rta (uninfected by lentivirus) were untreated (-D) or treated with doxycycline (+D) to induce replication. Expression of IFIT1 (A), IFIT3 (B) or ORF57 (C) was measured by immunoblotting. iSLK/Bac16 cells were infected with six independent lentivirus clones containing IFIT1 shRNA (shIFIT1) or control shRNA (sh C) and IFIT1 was measured by immunoblotting to assess efficacy of IFIT1 KD (D). TREx BCBL1 cells were infected with pooled IFIT1 shRNA lentivirus preparations or control lentivirus, and then mock-treated (-D) or treated with doxycycline (+D) to induce replication. Lysates were immunoblotted for IFIT1 (E) or IFIT3 (F). Lysates were also blotted with anti-ORF57 antibodies (G) or anti-K8.1 antibodies (H) to assess effects on KSHV lytic gene expression. Blots stripped and re-probed with anti-actin or anti-tubulin antibodies are shown below each panel as a loading control.
(TIF)

S7 Fig MonoQ Ion exchange chromatography (A) and S200 gel filtration chromatography (B) for RtcB enzyme preparation. Purification of raw RtcB was performed by Ion exchange (MonoQ) purification (S7A Fig) followed by S200 gel filtration (S7B Fig) with unsalted buffer, high salt buffer and buffer B. Purified RtcB was eluted and diluted to in buffer B with 0.5% Triton X-100, aliquoted and stored at -80°C.
(TIF)
