# Supplementary figures and images for: Human IFIT proteins inhibit lytic replication of KSHV: A new feed-forward loop in the innate immune system
Source: PLoS Pathog. 2019 Feb 19;15(2):e1007609. doi: 10.1371/journal.ppat.1007609 (PMC6396945; doi:10.1371/journal.ppat.1007609)

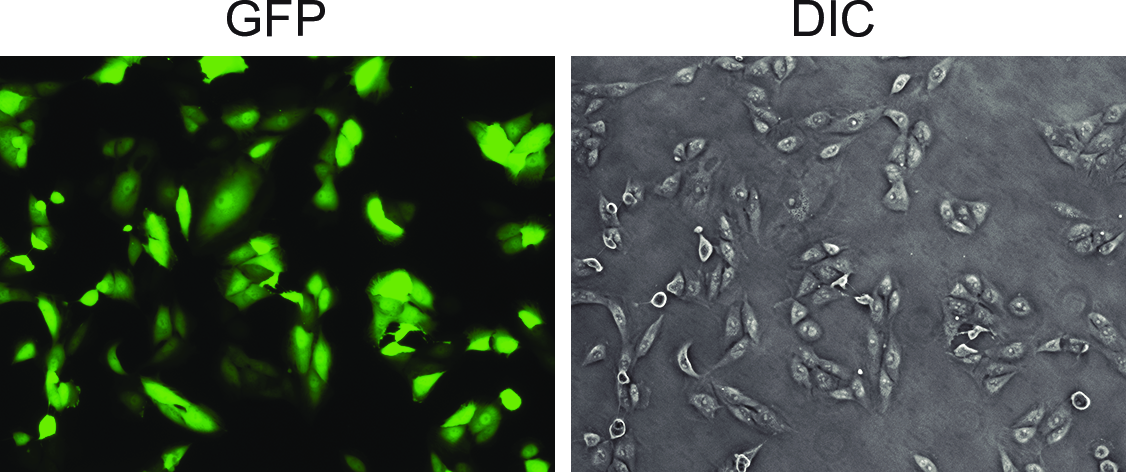

Supplement: S1 Fig — Images of GFP positive KSHV-infected iSLK cells were collected and analyzed with a Zeiss Observer D1 microscope system (left panel). Right panel shows phase contrast microscopy of cells in left panel. (TIF) [file ppat.1007609.s001.tif]

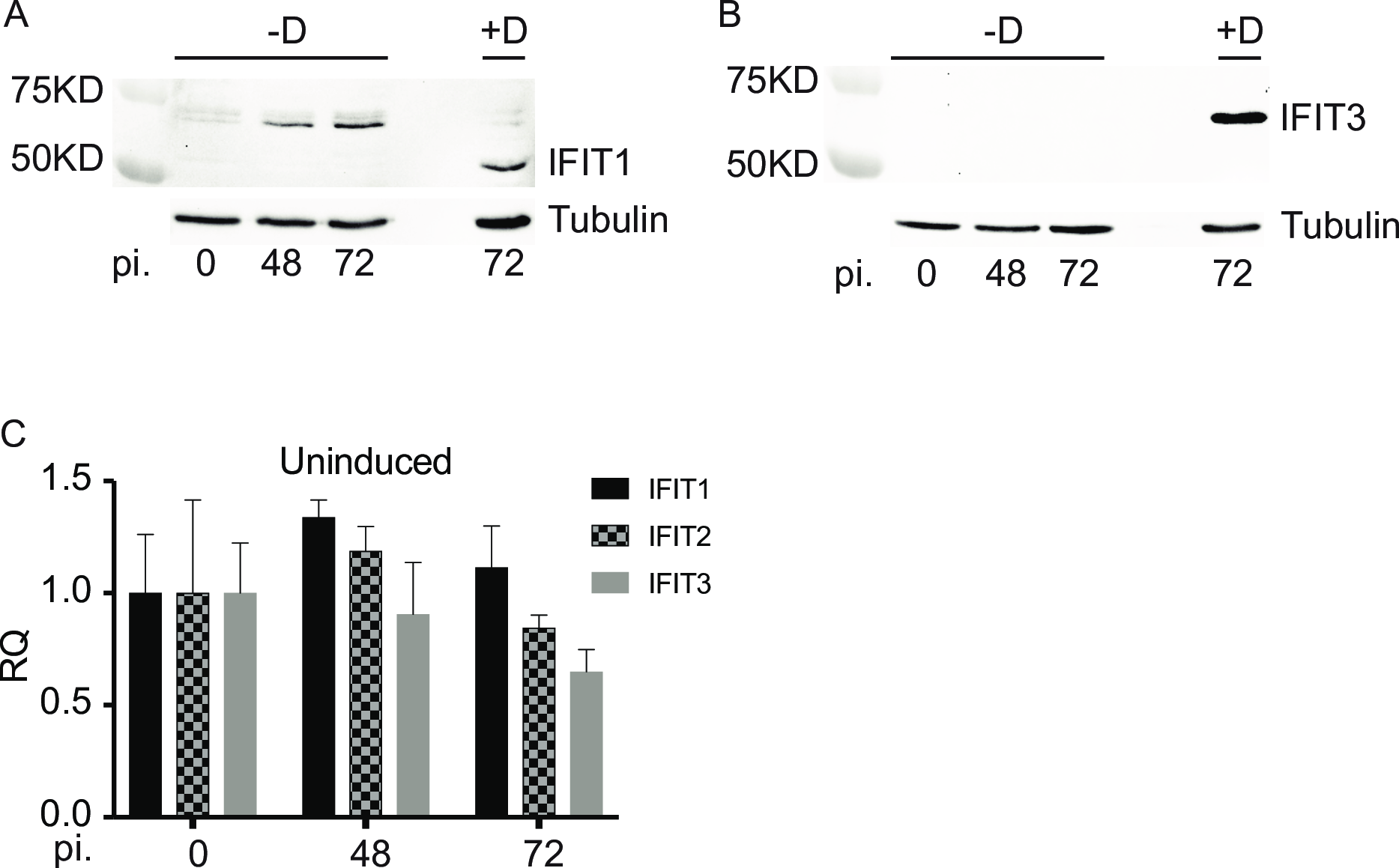

Supplement: S2 Fig — iSLK/Bac16 cells untreated (-D) with doxycycline were harvested at 48hr or 72hr post induction as shown. Immunoblotting of lysates from the cells was performed with anti-IFIT1 and anti-IFIT3 antibodies to measure IFIT1 (A) and IFIT3 (B) protein expression. Lysate from induced iSLK/Bac16 (KSHV-infected) at 72hr was used as a positive control in the rightmost lane (+D). Tubulin is shown as a loading control. qPCR was performed to measure IFIT RNA expression in the samples from 48hr and 72hr post induction as shown (C). (TIF) [file ppat.1007609.s002.tif]

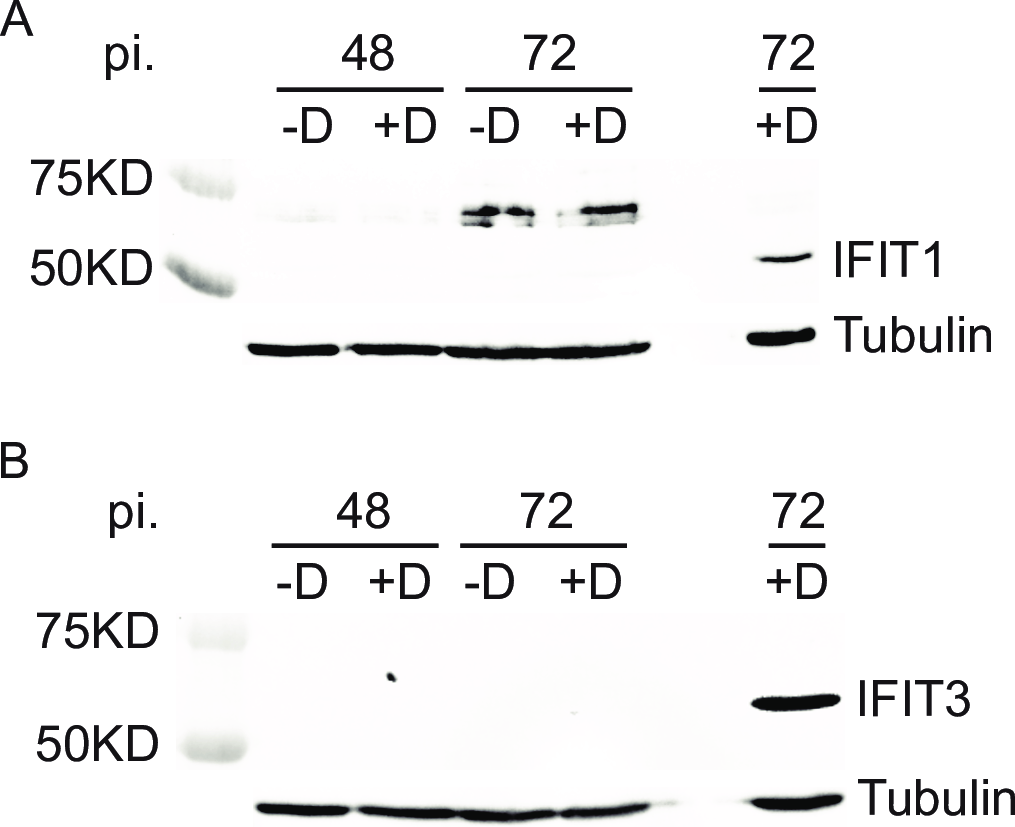

Supplement: S3 Fig — iSLK cells (without KSHV infection) were mock-treated (-D) or treated with doxycycline (+D). Cells were harvested at 48hr or 72hr post induction (pi.) as shown. Immunoblotting of lysates was performed with anti-IFIT1 and anti-IFIT3 antibodies to measure IFIT1 (A) and IFIT3 (B) protein expression. Lysate from doxycycline induced iSLK/Bac16 at 72hr was used as a positive control on right (72/+D). Tubulin is shown as a loading control. (TIF) [file ppat.1007609.s003.tif]

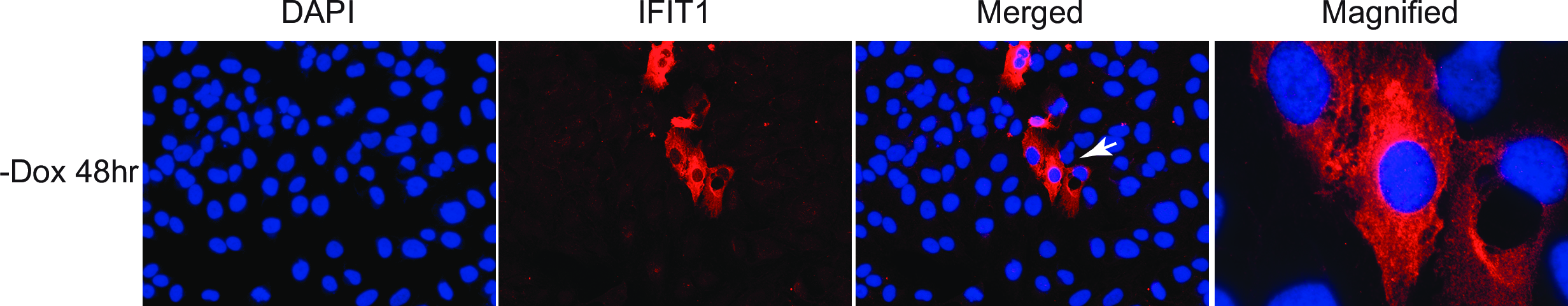

Supplement: S4 Fig — Cells were fixed at 48hr post-induction of lytic replication (pi) as shown. Cells were then stained for IFIT1 (Red). Arrows indicate magnified cells which are shown at right in the panel. DAPI staining of nuclei is shown in blue. (TIF) [file ppat.1007609.s004.tif]

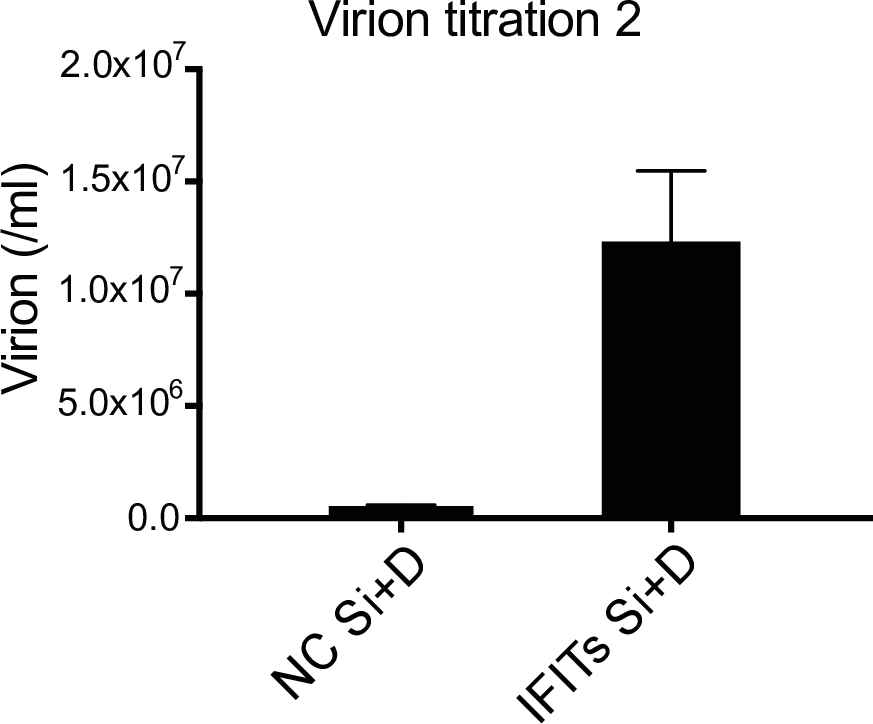

Supplement: S5 Fig — Virion titration 2.KSHV-infected iSLK cells were transfected with either control siRNA (NC Si) or a mixture of IFIT1, IFIT2 and IFIT3-specific siRNA (IFITs Si) and KSHV replication was induced by treatment with doxycycline. Supernatants from induced cells were used to infect 293T cells. Virus passage was quantitated by flow cytometry of GFP-positive 293T cells. Each transfection/induction was performed in triplicate and three replicate infections were performed with each supernatant. Error bars show SEM of titration from triplicate samples. (TIF) [file ppat.1007609.s005.tif]

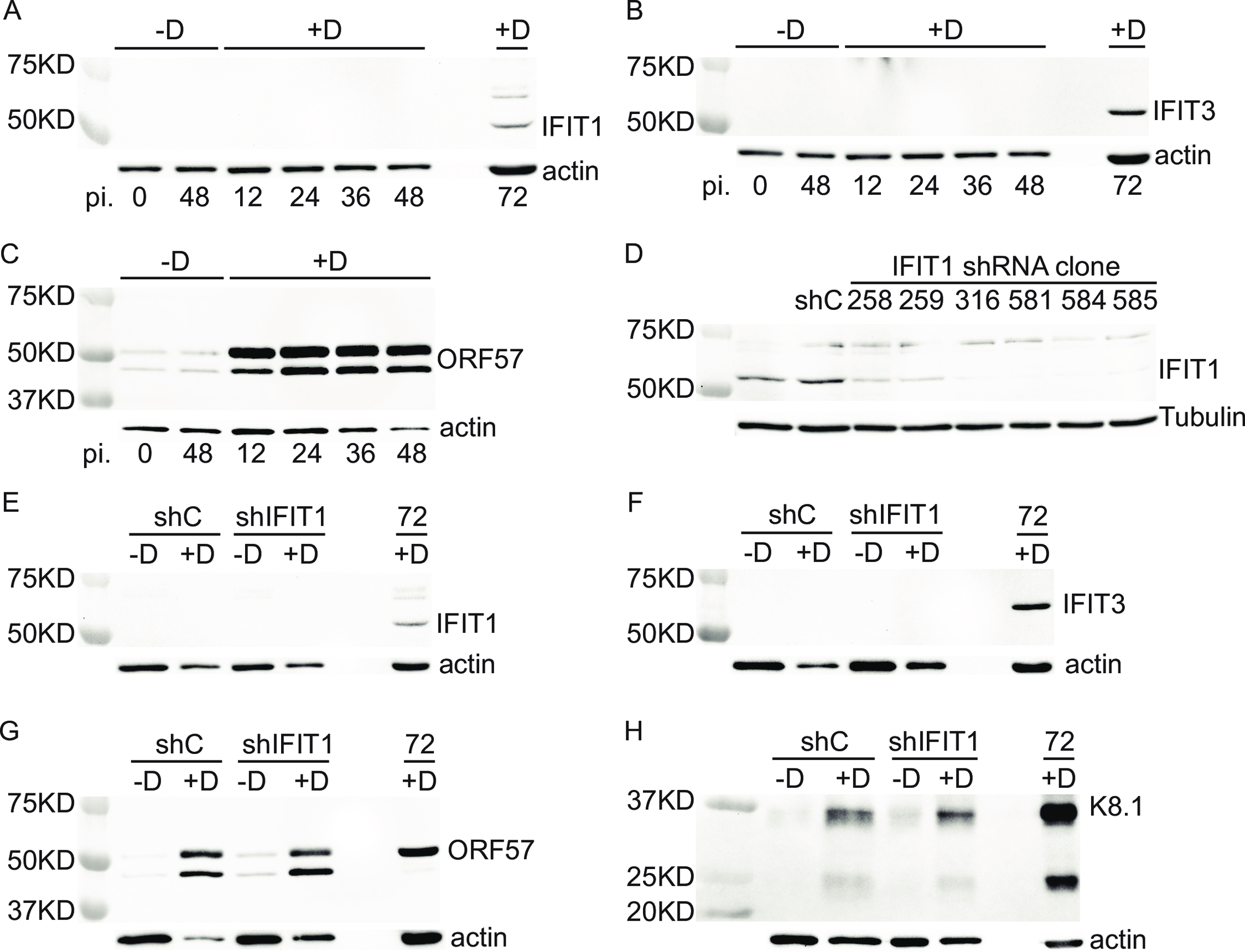

Supplement: S6 Fig — TRExBCBL1-Rta (uninfected by lentivirus) were untreated (-D) or treated with doxycycline (+D) to induce replication. Expression of IFIT1 (A), IFIT3 (B) or ORF57 (C) was measured by immunoblotting. iSLK/Bac16 cells were infected with six independent lentivirus clones containing IFIT1 shRNA (shIFIT1) or control shRNA (sh C) and IFIT1 was measured by immunoblotting to assess efficacy of IFIT1 KD (D). TREx BCBL1 cells were infected with pooled IFIT1 shRNA lentivirus preparations or control lentivirus, and then mock-treated (-D) or treated with doxycycline (+D) to induce replication. Lysates were immunoblotted for IFIT1 (E) or IFIT3 (F). Lysates were also blotted with anti-ORF57 antibodies (G) or anti-K8.1 antibodies (H) to assess effects on KSHV lytic gene expression. Blots stripped and re-probed with anti-actin or anti-tubulin antibodies are shown below each panel as a loading control. (TIF) [file ppat.1007609.s006.tif]

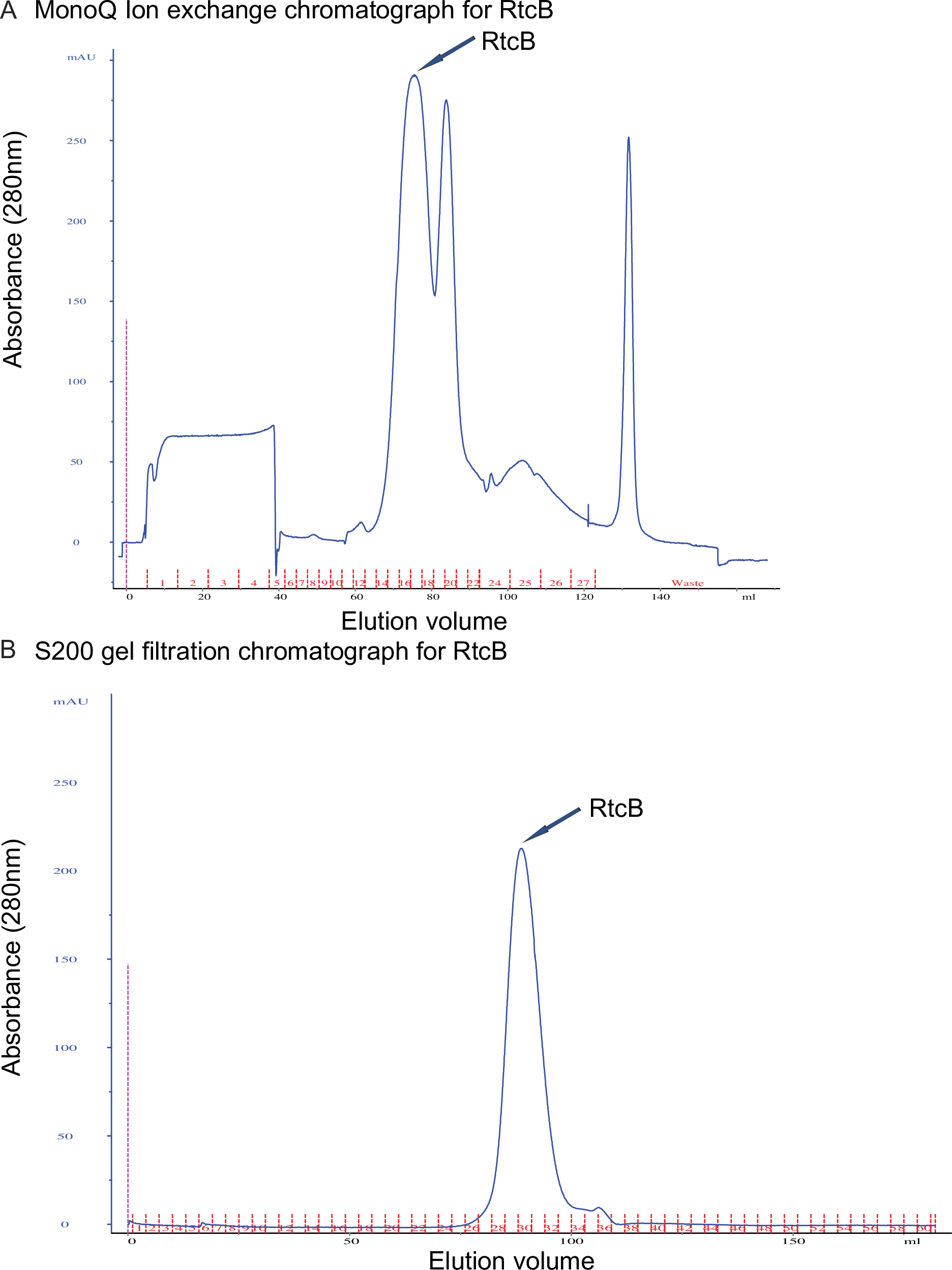

Supplement: S7 Fig — MonoQ Ion exchange chromatography (A) and S200 gel filtration chromatography (B) for RtcB enzyme preparation. Purification of raw RtcB was performed by Ion exchange (MonoQ) purification (S7A Fig) followed by S200 gel filtration (S7B Fig) with unsalted buffer, high salt buffer and buffer B. Purified RtcB was eluted and diluted to in buffer B with 0.5% Triton X-100, aliquoted and stored at -80°C. (TIF) [file ppat.1007609.s007.tif]
